# Molecular basis of genetic plasticity to varying environmental conditions on growing rice by dry/direct-sowing and exposure to drought stress: Insights for DSR varietal development

**DOI:** 10.3389/fpls.2022.1013207

**Published:** 2022-10-24

**Authors:** Suresh Kumar, Santosh Kumar, Gopala S. Krishnan, Trilochan Mohapatra

**Affiliations:** ^1^ Division of Biochemistry, ICAR-Indian Agricultural Research Institute, New Delhi, India; ^2^ Decode Genomics Private Limited, New Delhi, India; ^3^ Division of Genetics, ICAR-Indian Agricultural Research Institute, New Delhi, India; ^4^ Indian Council of Agricultural Research (ICAR), New Delhi, India

**Keywords:** planting method, reproductive-stage drought, genetic plasticity, ecological integrity, dry/direct-sown rice, water-productivity

## Abstract

Rice requires plenty of water for its cultivation by transplanting. This poses several challenges to its cultivation due to erratic rainfall resulting in drought, flood, and other abiotic stresses of varying intensity. Dry/direct-sown rice (DSR) has emerged as a water-saving/climate-smart alternative to transplanted rice (TPR). The performance of a rice cultivar on growing by different methods of planting under varying environmental conditions varies considerably. However, the molecular basis of the observed phenotypic plasticity of rice to varying environmental conditions is still elusive. Resilience to various environmental fluctuations is important to ensure sustainable rice production in the present era of global climate change. Our observations on exclusively up-regulated genes in leaf of Nagina 22 (N 22) grown by dry/direct-sowing and subjected to drought stress at panicle initiation stage (compared to that in leaf of IR 64), and another set of genes exclusively down-regulated in leaf of N 22 (compared to that in leaf of IR 64) indicate important roles of leaf in stress resilience. A large number of genes down-regulated exclusively in root of N 22 on dry/direct-sowing subjected to drought stress indicates a major contribution of roots in stress tolerance. The genes for redox-homeostasis, transcription factors, stress signaling, carbohydrate metabolism, and epigenetic modifications play important roles in making N 22 better adapted to DSR conditions. More importantly, the involvement of genes in rendering genetic plasticity to N 22 under changing environmental conditions was confirmed by reversal of the method of planting. To the best of our knowledge, this is the first report on decoding the molecular basis of genetic plasticity of rice grown by two different methods of planting subjected to drought stress at the reproductive stage of plant growth. This might help in DSR varietal development program to enhance water-productivity, conserve natural resources, and minimize the emission of greenhouse gases, thus achieving the objectives of negative-emission agriculture.

## Introduction

The global population is predicted to cross 9 billion by the year 2050, which would result in an increased demand for food by 70% (Bruinsma, 2009; Kumar, 2013). To feed the burgeoning global populations, we would require producing more food and livelihood opportunities from the continuously diminishing per capita availability of land and water. More importantly, providing ample food to the ever-growing population is the first part of the challenge; a more important challenge is to produce them safely and sustainably (Kumar, 2012). Improving resilience to environmental stresses is crucial to increase crop yield and ensuring sustainable food production, particularly in the present era of global climate change. Rice is one of the most important food crops which fulfil the caloric mainstay for half of the global population (Seck et al., 2012). The water-loving nature of rice requires plenty of water for its irrigation/cultivation by transplanting. Globally, three-fourths of rice is grown by transplanting (Rao et al., 2007); however, transplanted rice (TPR) requires lots of water for continuous irrigation (resulting in limited availability of water for irrigation of other crops, particularly in the year of drought/low rainfall). TPR has been associated with the emission of greenhouse gases and its cultivation is considered to be an input-intensive practice. TPR requires 2000 to 5000 litres of water for every kilogram of rice grain produced, depending on the irrigation method and the prevailing environmental conditions (Bouman, 2009; Seem and Kumar, 2021). A major proportion of the irrigation water is lost from flooded rice fields due to evaporation, transpiration and deep percolation, resulting in low water-productivity (Kumar, 2021). Puddling, a necessary step in the transplantation of rice, has been reported to adversely affect the performance of the succeeding crop due to poor crop establishment (Rahmianna et al., 2000), soil physical properties  (Gathala et al., 2011), growth of root because of subsurface compaction of soil and formation of hardpan at shallow depth (Kalita et al., 2020). Moreover, flood-irrigation of rice (continuously flooded rice field) is one of the main reasons for the emission of anthropogenic greenhouse gases (GHG). Thus, both excessive (flood) and scarcity (drought) of water in rice fields adversely affect the overall yield of the crop. Because of the above-mentioned disadvantages of TPR, emphasis is being given to shifting from TPR to dry/direct-sown rice (DSR).

DSR is considered to be an efficient, resource-conserving technology which reduces the requirements of tillage and labor needed particularly for puddling and transplantation of rice (Chakraborty et al., 2017). For DSR, seeds are sown directly in dry/non-puddled soil (Liu et al., 2015), which not only minimizes the input costs but also the labor requirements, if herbicide tolerant DSR cultivar is developed/utilized. In the areas with limited availability of fresh water and labor, adoption of DSR with zero or minimum tillage would reduce the cost of rice cultivation (Rao et al., 2017). The above-mentioned advantages of DSR are attracting farmers, researchers, ecologists and policymakers to work towards shifting from TPR to DSR. However, the constraints in achieving optimal growth and productivity of DSR include poor germination, stand establishment, nutrient uptake, weed infestation and occurrence of abiotic stresses like drought (Liu et al., 2014; Palanog et al., 2014; Kumar et al., 2022a). Even then, DSR is gaining popularity among farmers mainly because of the low input costs and its less water-demanding nature (Rao et al., 2007).

DSR faces the challenges of reduced nutrient uptake, particularly nitrogen, phosphorus, iron, and zinc due to aerobic conditions which affect tillering, leaf area index, photosynthesis, plant growth, and productivity of the crop. In addition, increased risks of weed and nematode infestations are some of the major biotic constraints in the adoption of DSR (Rao et al., 2017; Sagare et al., 2020). Global climate change, erratic rainfall, fluctuating temperature, frequent drought stress, etc. pose serious concerns for sustainable rice cultivation (Nawaz et al., 2022). Nevertheless, DSR could be a promising, economically sustainable technology (Liu et al., 2014). Though switching to DSR has become a necessity of the day to save water, only a limited number of cultivars exhibit comparable yield under DSR (drought-prone rainfed) conditions. Currently, most of the cultivars used for DSR are the landraces with superior early/seedling growth, multiple abiotic stress tolerance, and better yielding potential under rainfed conditions (Mahajan et al., 2018). With the use of improved cultivars, weed management practices, and protective measures, DSR can provide a yield comparable to TPR (Zhao et al., 2007). Improvement variety for DSR conditions can be achieved by combining the traits for yielding potential and adaptability to abiotic/biotic stresses (Subedi et al., 2019). However, information on the molecular basis of the genetic plasticity of rice for better performance under varying environmental conditions that persist in direct-sown and transplanted rice fields is still elusive.

Drought stress is one of the major constraints of rice cultivation under rainfed conditions. Moreover, drought stress at the reproductive (panicle initiation to grain filling) stage has considerable adverse effects on crop productivity (Anantha et al., 2016; Kumar et al., 2022a). Drought and the associated abiotic stresses cause multiple impairments including metabolic disorders, cell injury through the generation of reactive oxygen species (ROS), and increased cellular temperature. All of these result in progressive oxidative damage leading to cell death (Farooq et al., 2008; Palanog et al., 2014; Sasi et al., 2021). Rice is more sensitive to reduced soil moisture content (SMC) compared to other cereals like maize and wheat. Despite these facts, the majority of rice is planted with high-yielding, drought-sensitive rice cultivars like IR 64. With increasing pressure on food grain production and diminishing availability of fresh water, various water-saving techniques to enhance water-productivity are required to be adopted in the cultivation of rice (Ishfaq et al., 2020; Kumar, 2021). Therefore, the focus is now shifting to the adoption of DSR having comparable yield even under intermittent drought stress of varying intensity (Seem and Kumar, 2021). TPR is vulnerable to water-deficit stress, particularly at the flowering/reproductive stage, resulting in spikelet sterility and yield losses. Delay in transplanting due to water management-related issues results in less tillering and reduced yield. On the other hand, the DSR cultivar has innate drought as well as other abiotic stress tolerance and better survival during a dry period with the yielding potential comparable to TPR. Moreover, DSR requires only one-third to one-fourth of the water required for TPR. Replacing TPR with DSR would not only enhance water-productivity but will also help in the realization of the slogan “*Per Drop More Crop*” focused on saving/conserving water for better ecological efficiency and integrity (Kumar, 2021).

Nagina 22 (N 22), a tall, deep-rooted, drought and heat-tolerant *aus* rice (Jagadish et al., 2010), is characterized by its ability to perform better in rain-fed, heat-prone areas. Its early maturity (90–95 days) makes it one of the suitable cultivars for DSR (Vikram et al., 2011). It performs well under both DSR and TPR conditions. In contrast, IR 64 is a high-yielding, semi-dwarf, lowland *indica* cultivar (Uga et al., 2013) having a relatively shallow root system (Gowda et al., 2012; Shrestha et al., 2014; Mackill and Khush, 2018). However, it is more sensitive to drought stress at the reproductive stage resulting in a considerable reduction in yield (Anantha et al., 2016). Its yielding potential was reported to be considerably low under aerobic (upland/dry/direct-sown) conditions (Zhao et al., 2010).

Domestication of crop plants and development of high-yielding cultivars resulted in reduced drought tolerance and more fertilizer-responsive genotype due to significant genetic changes. Plants use a variety of physiological, biochemical, and molecular machinery to protect them from abiotic stresses. N 22 has been used as a donor in breeding drought-tolerant rice cultivars (Lenka et al., 2011; Vikram et al., 2011). Several transcriptome analyses have been reported for expression profiling of genes under drought stress in rice (Lenka et al., 2011; González-Schain et al., 2015; Shankar et al., 2016; Sinha et al., 2018). Some of the quantitative trait loci (QTLs) for DSR-favoring traits like early seedling emergence, vegetative vigor, root architecture, plant height, and biomass production have been reported (Sandhu et al., 2015). Moreover, inter-cross populations with nutrient-efficient and high-yielding potential have also been identified (Subedi et al., 2019). However, the candidate genes and their role in the acclimatization of rice to the varying environmental conditions in direct-sown and transplanted rice fields have not yet been known. A better understanding of the molecular basis of efficient root system architecture (RSA), nutrient use-efficiency, and grain yield might help develop rice varieties with better adaptability to DSR conditions and enhanced productivity. This might help improve DSR varieties towards mitigating the effects of GHG emissions and global climate change for better ecological efficiency/integrity. Our recent transcriptome analysis revealed the genes/pathways responsible for better performance of rice grown by direct-sowing (Kumar et al., 2022a). However, no report is available on a comprehensive analysis of the genes responsible for the genetic plasticity of rice to the varying environmental conditions observed in TPR and DSR fields, particularly on the occurrence of a common/frequent (drought) abiotic stress. Such information is necessary to have better insights into the adaptive mechanisms involved in making DSR cultivars better performers in rainfed/drought-prone areas. Therefore an attempt was made to unravel the genes involved in the genetic plasticity of rice to different methods of planting (transplanting and dry/direct-sowing) on subjecting to reproductive stage drought using a pair of popular *Indica* rice cultivars. More importantly, we confirmed the involvement of genes in the genetic plasticity of N 22 by changing the method of planting (plants continuously grown by direct-sowing were shifted to transplanting and vice versa) and assessing the expression of the genes. Thus, we decoded and verified the molecular basis of genetic plasticity/better performance of N 22 on dry/direct-sowing under unfavourable environmental conditions, particularly the occurrence of reproductive stage drought.

## Materials and methods

Mature seeds of two rice cultivars (Nagina 22, drought and heat tolerant; IR 64, sensitive to reproductive stage drought) were grown by both direct-sowing and transplanting continuously for five consecutive generations/years (**Supplementary Method S1A**). For TPR, seedlings were raised in a nursery, followed by uprooting the 25-days-old seedlings and transplanting them in pots (12″ diameter) containing puddled soil. For DSR, mature seeds were directly sown in pots filled with unirrigated soil (moisture content~9% by weight, compared to~24% in the soil used for TPR). Three plants were maintained in each pot. In the sixth year, the seeds collected from direct-sown drought-treated plants were used to grow rice by transplanting (D→T), and the seeds collected from transplanted drought-treated plants were used to grow rice by direct-sowing (T→D) (**Supplementary Method S1B**). The plants were grown under natural conditions in a net-house from July−October at the experimental farm of ICAR-Indian Agricultural Research Institute, New Delhi, India.

### Drought stress treatment

The pots, each containing three plants, were divided into two sets each of eight pots. One set of the pots was grown as control (irrigated on an alternate day with tap water), while the other set was maintained for drought stress treatment at the reproductive (initiation of flowering) stage of plant growth by withholding irrigation. The DSR pots were maintained with life-saving irrigation in absence of the seasonal rainfall. Likewise, one set of DSR pots was grown as control (with life-saving irrigation), while the other set was imposed with drought stress at the reproductive stage. The plants were subjected to drought stress by withholding irrigation for 4−5 days just before panicle initiation (65 days after transplanting of N 22). The level of drought stress was assessed by measuring soil moisture content (dropped down to ~6% by weight) and relative water content (dropped to~58%) of leaves, which was~24% (SMC) and~72% (RWC) in the control pots and plants, respectively. The effects of reduced SMC and RWC in leaf could be visualized by observing the morphology of the drought-treated plants (**Supplementary Figure S1**). The leaf and root tissues were collected in eight replications from the rice plants, grown by different methods of planting under control as well as drought conditions, in liquid nitrogen for molecular analysis.

### Estimation of soil moisture content and relative water content

Soil moisture content (SMC) was estimated by the gravimetric method by collecting soil samples from 5 cm depth in the pots. The soil samples were kept in Petri-plate after recording the initial weight, followed by placing them in an oven for drying at 60 °C until a constant weight was achieved. SMC was calculated using the formula:


SMC (%) = [(weight of wet soil) - (weight of dry soil)]/(weight of dry soil ×100)

Relative water content (RWC) of leaves was estimated by collecting fresh tissues from the upper half of leaves, cutting them into 1.0 cm pieces and keeping them in a pre-weighed Petri plate. Fresh weight (FW) of the leaf tissues was recorded immediately, and distilled water was poured into the Petri plate, covered with a lid, and incubated at room temperature for 4 h. Turgid weight (TW) of the leaf tissue was recorded, the tissues were dried by blotting them between layers of paper towels and finally dried in an oven at 60 °C until a constant weight of the tissue was achieved. The dry weight (DW) of the sample was recorded, and RWC was calculated by using the formula:


RWC (%) = [(FW - DW)/(TW - DW)] ×100

### Assessment of root architecture and agronomic performance

To assess the architectural difference in root of the plants grown by different methods of planting at the seedling as well as reproductive stage, roots from representative plants were cut at the root–shoot junction, washed with water, and spread in a root positioning tray (30 × 40 cm) filled with water up to 1 cm depth. The roots were scanned at 600 dpi on a grey scale using a desktop scanner (Epson 100XL flatbed scanner). To assess the effects of different methods of planting on the agronomic performance of rice, the number of tillers, number of panicles, number of grains, and test weight of seeds were counted/measured in three replications. The number of tillers per plant was recorded at the age of 50 days for N 22 and 60 days for IR 64.

### Estimation of protein content in grains grown by different methods of planting

To estimate total protein (in terms of nitrogen) content in mature/dehusked seeds of rice, 100 mg seeds were crushed into a fine powder and added into a Kjeldahl digestion flask along with 10 ml conc. sulfuric acid. To improve the rate and efficiency of digestion, 2 g of potassium sulfate and copper sulphate mixture (10:1 ratio) was added to each tube. The digestion flask was heated to~400 °C on a heating block for 180 min and digestion was stopped when the sample became transparent with a slight blue color. On completion of digestion, the sample was allowed to cool down to room temperature and diluted by adding 25 ml of water. The digested/diluted extract was transferred to a distillation unit, added with 50 ml of sodium hydroxide (40% solution) slowly along the wall of the digestion flask to neutralize the sample and convert ${\text{NH}}_{4}^{+}$ into NH_3_. The emitted NH_3_ on distillation was captured in 25 ml of boric acid (4%) solution containing 6 drops of Mixed indicator. The reaction of NH_3_ with boric acid converted the solution from red-violet to green color. The condenser was rinsed with water to make sure that all the ammonia has been recovered. The distillate was titrated with 0.1 N HCl until the solution became slightly violet indicating the end-point.

### RNA isolation, cDNA library preparation and sequencing

Total RNA was isolated in six biological replications from leaf and root tissues using TRIzol reagent. The RNAs were pooled (3 + 3) in two groups for each of the tissue samples (**Supplementary Method S1C**). A total of 48 (32 + 16) cDNA libraries were prepared from the samples collected during two (5^th^ and 6^th^) years of the experiment following the procedure described earlier (Kumar et al., 2021a). The libraries were sequenced on Illumina HiSeq 2500 platform using PE-150 chemistry. Raw sequence data were submitted to the NCBI under the BioProject Submission IDs: PRJNA805549, PRJNA833055, and SUB11354353, which were used for bioinformatic analysis.

### Quality check and RNA-seq data analysis

The quality of the raw data was assessed with the help of FastQC 0.11.7 (http://www.bioinformatics.babraham.ac.uk/projects/fastqc). Adapter contamination and low-quality reads (<Phred33) were removed using Trimmomatic 0.36 (Bolger et al., 2014). The high-quality reads thus obtained were mapped to the rice reference genome (RGAP, http://rice.plantbiology.msu.edu) using HISAT2 (2.1.0) pipeline (Pertea et al., 2016) and assembled using StringTie package with default parameters to construct unique transcript sequences. The number of mapped clean reads for each gene was counted and normalized in terms of reads per kilobase per million (RPKM). The differentially expressed genes (DEGs) were analyzed using DESeq2 (V 1.20.0) package of the R program (Love et al., 2014). False discovery rate (FDR) ≤0.1, *P*<0.05, and log2 FC ≥ ± 2 were used as the threshold to judge the significance of the difference.

### Gene ontology analysis

Gene ontology (GO) enrichment analysis of the DEGs was performed using AgriGO v2 (http://bioinfo.cau.edu.cn/agriGO; Du et al., 2010) and ShinyGO v0.75 software (http://bioinformatics.sdstate.edu/go) with false discovery rate (FDR) <0.05. The analyses identify enriched/under-represented GO terms by comparing a list of query genes and their corresponding GO terms (extracted from the Rice Genome Annotation Project database) with a background population list from which the query list was derived. T-tests were carried out to identify a significant difference between the query genes and all other background genes.

### Validation of DEGs by RT-qPCR

To verify the RNA-seq results, the expression level of some of the randomly selected genes playing important roles in the adaptation of rice to direct-sown and drought conditions were validated by RT-qPCR. The total RNAs isolated from leaf and root tissues of N 22 and IR 64 (grown by transplanting and direct-sowing) were subjected to DNase I treatment, followed by reverse transcription using superscript II (Invitrogen). RT-qPCR validation of the selected genes was performed in three biological and three technical triplications using SYBR Green PCR Master Mix kit (Applied Biosystems, CA, USA) following the manufacturer’s instructions using QIAquant 96 5plex machine (Qiagen, Germany). Details of the primers used for RT-qPCR validation are listed in **Supplementary Table S1**. RT-qPCR was performed in a 10 µl reaction mix and the thermal cycler was programmed for initial denaturation at 95°C for 3 min, followed by 40 cycles each of denaturation at 94°C for 10 s, annealing at 60°C for 15 s, and extension at 72°C for 30 s. Data collection was set at the end of every extension step and the data was used for melt curve analysis. Relative gene expression was determined using the 2^−ΔΔCt^ method. Actin and tubulin were used as internal reference genes.

### Statistical analysis

The experiments were carried out in three or more replications. Statistical analysis was performed by analysis of variance (ANOVA), *Post hoc* Tukey test or Duncan’s multiple range test (DMRT) at *P* ≤ 0.05 were used to compare the means of treatments. For RT-qPCR analysis, the relative expression level represents the fold change in expression of the target gene. The error bars represent the standard deviation ( ± SD).

## Results

### Root morphology and agronomic performance of rice on growing by different planting methods

When the rice was grown by dry/direct-sowing, a lower SMC (~12%) was recorded in the soil during the initial 7 days affecting germination and seedling establishment. The reduced SMC resulted in a 40% reduction in the germination of the seeds of IR 64, but the reduction in germination was observed to be only 20% in the case of N 22, while 100% germination was recorded in the wet/watered soil. At the reproductive stage of growth, the SMC was recorded to be 9% in the direct-sown pots compared to 24% SMC in the well-watered transplanted pots. At the reproductive stage of plant growth, the RWC in leaf of IR 64 grown by direct-sowing was recorded to be 63±1%, while it was slightly higher (66±1%) in leaf of N 22. RWC of IR 64 and N 22 leaf was recorded to be 71±2% when they were grown by transplanting. Drought stress treatment was performed by withholding irrigation (until SMC dropped down to ~6%) which resulted in a reduction of RWC (58±1%) in leaf of IR 64 compared to 61±1% RWC in leaf of N 22. Moreover, the symptoms of drought stress (rolling/wilting of leaves) were observed on the plants (**Supplementary Figure S1**).

**Figure 1:**
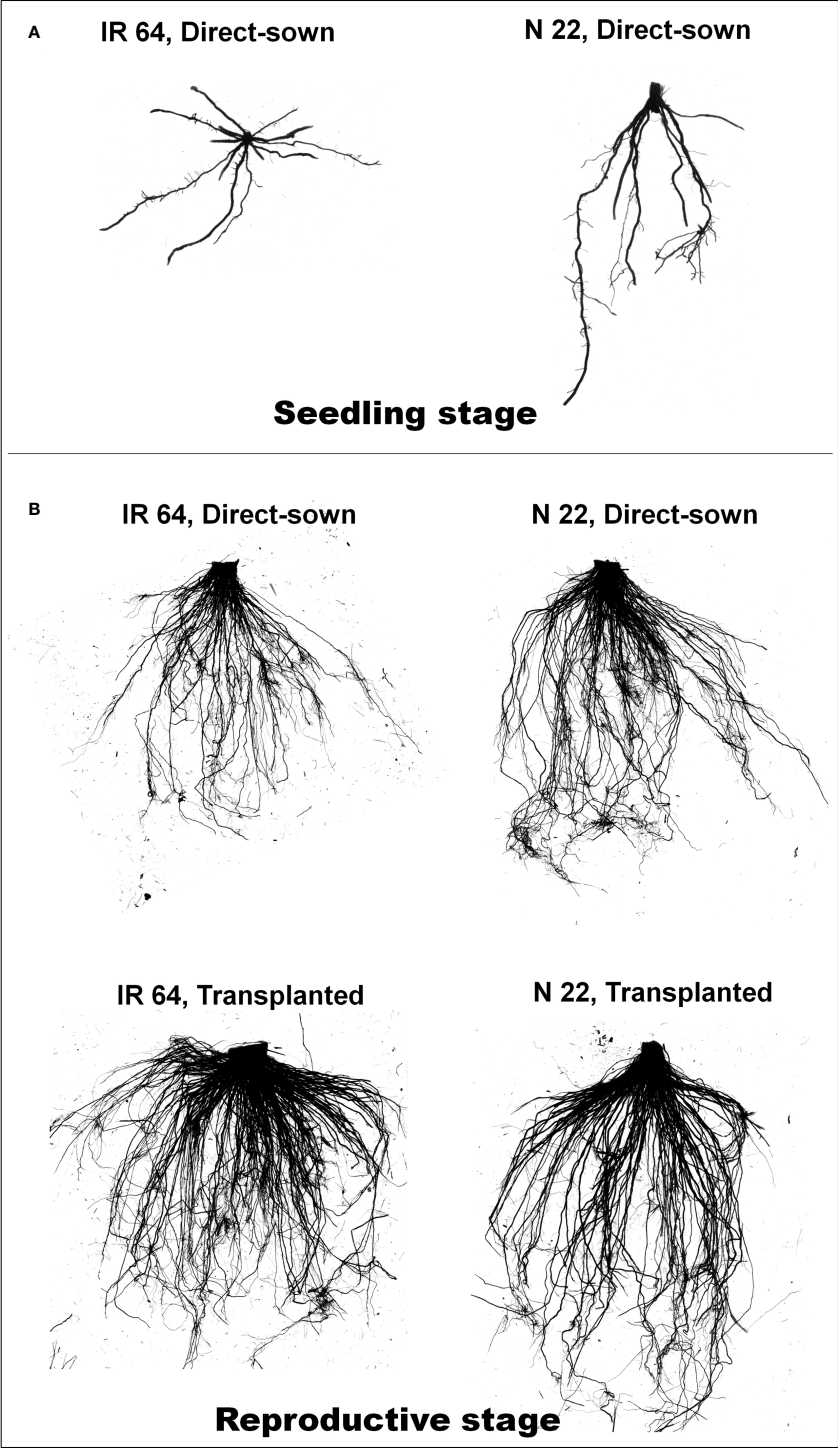
Representative profiles of root architecture of rice grown by different methods of planting. **(A)** Root of the seedlings grown by direct-sowing, **(B)** root of a plant [IR 64 and Nagina 22 (N 22)] at reproductive stage grown by different methods of planting (n=3).

A significant difference in root morphology of the rice cultivars at the seedling stage was observed when they were grown by direct-sowing. Horizontal growth of roots was observed in the case of IR 64 when grown by direct-sowing, while vertical growth of the roots was observed in the case of N 22 seedlings grown by direct-sowing (**Figure 1A**). When seedlings were grown in the nursery (ample availability of water) for transplanting, no significant difference in root system architecture was observed between IR 64 and N 22 (**Supplementary Figure S2**). At the reproductive stage of growth, fewer roots were observed when IR 64 was grown by direct-sowing, but dense horizontal roots were observed when grown by transplanting (**Figure 1B**). N 22 showed deeper roots when grown by direct-sowing. Not much difference in root system architecture (RSA) was observed when N 22 was grown by transplanting (**Figure 1B**).

**Figure 2:**
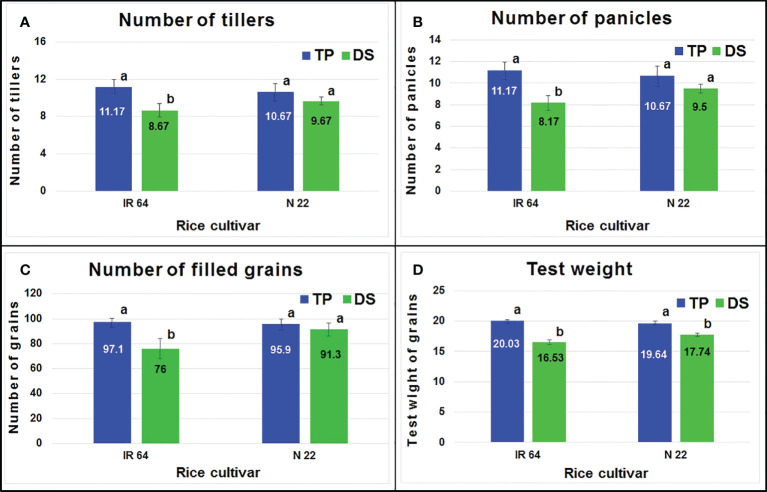
Agronomic performance of rice grown by different methods of planting. **(A)** Number of tillers per plant, **(B)** number of panicles per plant, **(C)** number of filled grains per panicle, **(D)** test-weight (g) of (1000) seeds. TP= transplanted, DS= direct-sown. Data present mean value (n=3). Mean followed by different lower-case letters are significantly different (P<0.05). The error bars represent the standard deviation ( ± SD).

Agronomic performance of the rice cultivars under direct-sowing and transplanting was assessed in terms of the number of tillers which showed a significant reduction in IR 64 on direct-sowing, while only a non-significant reduction was observed in the case of N 22 (**Figure 2A**). Similarly, a significant reduction in the number of panicles was observed in the case of IR 64, but only a non-significant reduction was observed in the case of N 22 (**Figure 2B**). The number of grains (filled) was observed to be significantly lesser when IR 64 was grown by direct-sowing, while it was comparable in the case of N 22 when grown either by direct-sowing or transplanting (**Figure 2C**). Though a significant reduction in test weight of the seeds was observed when IR 64 was grown by direct-sowing, only a small decrease in test weight was observed in the case of N 22 when grown by direct-sowing (**Figure 2D**).

**Figure 3:**
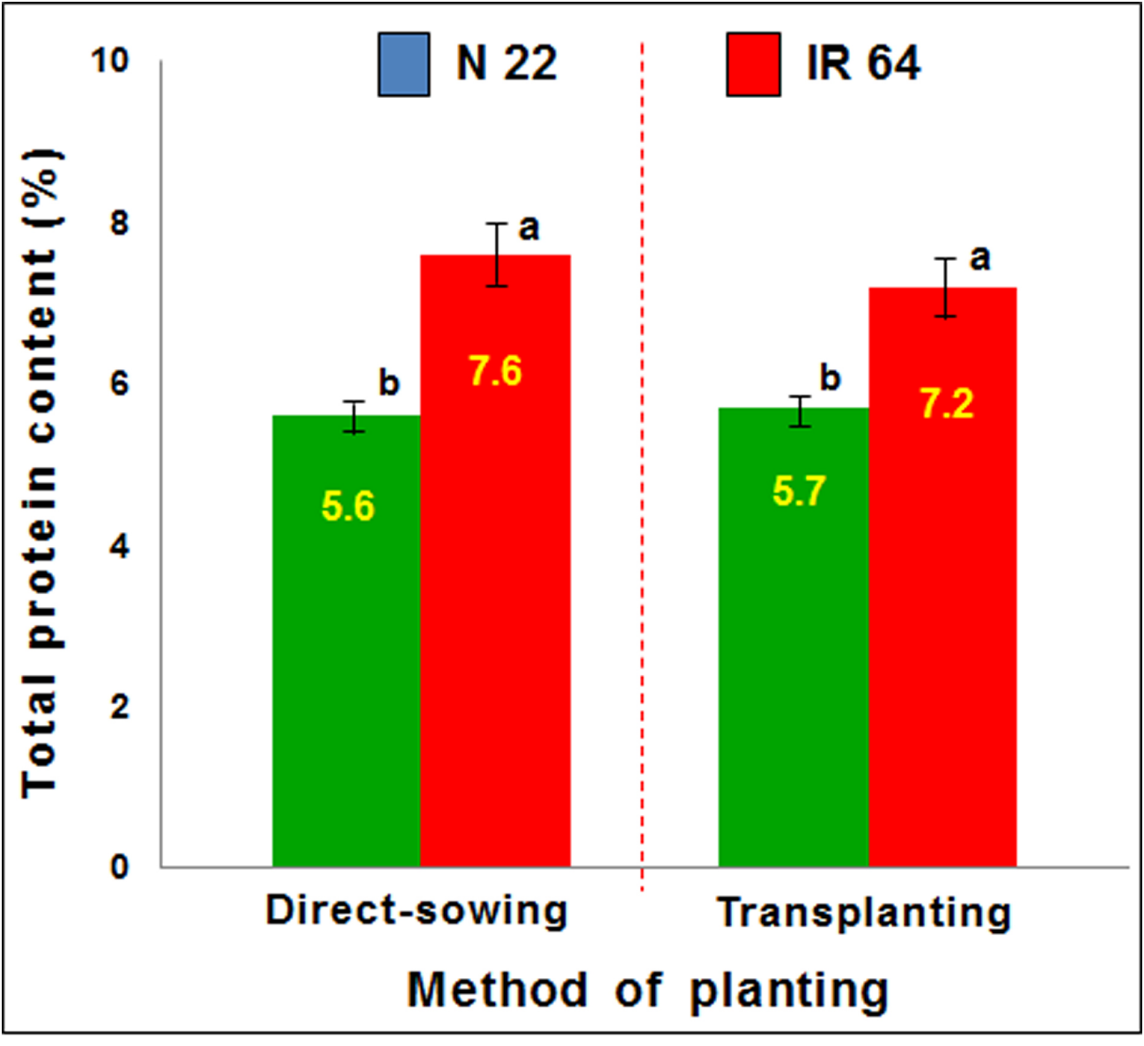
Effect of the methods of planting (direct-sowing and transplanting) on protein content in mature seeds. Data represent the mean ± SD (n = 3). Mean values followed by different lowercase letters (a, b) are significantly different (P ≤ 0.05).

The performance of the rice cultivars on different methods of planting was also assessed in terms of protein yield. Total protein content in mature seeds showed a minor increase in the seeds of IR 64 when grown by direct-sowing, compared to that observed in the seeds grown by transplanting. This increase in protein content corresponded with the reduced grain yield. In the case of N 22, no significant change in protein content was observed when it was grown either by direct-sowing or transplanting (**Figure 3**). However, a significant reduction in protein yield (protein content × grain yield) was observed in the case of IR 64 when grown by direct-sowing and subjected to drought stress which caused a significant decrease in grain yield.

### Library preparation, sequencing and mapping on reference genome

To identify the genes involved in genotypic plasticity of N 22 to direct-sown/transplanted conditions and under reproductive-stage drought stress, RNA-seq data were used for comparative analysis. The libraries prepared in replications for root and leaf tissues from the rice cultivars grown by direct-sowing/transplanting under control/drought stress, and then by changing the method of planting, were sequenced using PE-150 chemistry. A total of 11171 million reads, with an average of 23 million reads for each tissue sample, were generated. Reference-based mapping of the RNA-seq data on reference rice genome (TIGR v7) showed sufficiently high (89.89%) mapping efficiency (**Table 1**).

**Table 1:** Summary of RNA-seq data mapping statistics for leaf and root tissues from rice cultivars (IR 64 and Nagina22) grown by direct-sowing and transplanting, and then by changing the method of planting (Transplanted→Direct-sown, T→D; Direct-sown→Transplanted, D→T) under control conditions and imposition of drought stress at reproductive-stage.

Sample ID	Replication	Description	Total reads	Trimmed reads	Mapping efficiency (%)
ILTC1	1	IR 64, Leaf, Transplanted, Control	22581026	17292473	86.11%
ILTC2	2		20548142	18102886	88.38%
ILTD1	1	IR 64, Leaf, Transplanted, Drought	21659131	16224696	86.62%
ILTD2	2		25471309	18657858	89.29%
ILDC1	1	IR 64, Leaf, Direct-sown, Control	27273614	18727297	91.78%
ILDC2	2		22581026	17292473	91.83%
ILDD1	1	IR 64, Leaf, Direct-sown, Drought	22656359	17292473	94.13%
ILDD2	2		18285575	16713784	91.11%
IRTC1	1	IR 64, Root, Transplanted, Control	21945889	18114022	88.34%
IRTC2	2		24056316	21828283	90.47%
IRTD1	1	IR 64, Root, Transplanted, Drought	27056316	24828283	90.47%
IRTD2	2		23656359	19292473	94.13%
IRDC1	1	IR 64, Root, Direct-sown, Control	20548142	15102886	88.38%
IRDC2	2		16285575	14713784	86.11%
IRDD1	1	IR 64, Root, Direct-sown, Drought	21123405	18763808	90.8%
IRDD2	2		25328497	21851122	88.61%
NLTC1	1	N 22, Leaf, Transplanted, Control	26328497	22851122	89.61%
NLTC2	2		23223406	19763808	93.80%
NLTD1	1	N 22, Leaf, Transplanted, Drought	25471309	18657858	89.29%
NLTD2	2		22123405	17763808	92.80%
NLDC1	1	N 22, Leaf, Direct-sown, Control	25776457	19364537	90.71%
NLDC2	2		23945886	18114022	88.34%
NLDD1	1	N 22, Leaf, Direct-sown, Drought	25471309	18657858	89.29%
NLDD2	2		20659131	17224696	88.62%
NRTC1	1	N 22, Root, Transplanted, Control	21659131	16224696	86.62%
NRTC2	2		16285575	14713784	86.11%
NRTD1	1	N 22, Root, Transplanted, Drought	27273614	19727297	88.78%
NRTD2	2		22659131	18224696	86.62%
NRDC1	1	N 22, Root, Direct-sown, Control	22581026	17292473	90.83%
NRDC2	2		25056316	21828283	90.47%
NRDD1	1	N 22, Root, Direct-sown, Drought	27273614	21727297	90.78%
NRDD2	2		22123405	17763808	90.80%
ILDT→D1	1	IR 64, Leaf, Drought, Transplanted→ Direct-sown	25471309	18657858	91.29%
ILDT→D2	2		20659131	16224696	90.62%
IRDT→D1	1	IR 64, Root, Drought, Transplanted→ Direct-sown	25776457	20548142	89.71%
IRDT→D2	2		19548142	17102886	88.38%
ILDD→T1	1	IR 64, Leaf, Drought, Direct-sown→Transplanted	28603641	21123405	92.65%
ILDD→T2	2		22123405	17763808	93.8%
IRDD→T1	1	IR 64, Root, Drought, Direct-sown→Transplanted	23112742	18114022	89.33%
IRDD→T2	2		19285575	16713784	88.11%
NLDT→D1	1	N 22, Leaf, Drought, Transplanted→ Direct-sown	22656359	17292473	92.13%
NLDT→D2	2		25656254	19273476	91.13%
NRDT→D1	1	N 22, Root, Drought, Transplanted→ Direct-sown	25776457	19763808	90.71%
NRDT→D2	2		20945886	17114023	88.34%
NLDD→T1	1	N 22, Leaf, Drought, Direct-sown→Transplanted	28328497	24851122	91.61%
NLDD→T2	2		22273514	17727254	91.78%
NRDD→T1	1	N 22, Root, Drought, Direct-sown→Transplanted	26536698	22083513	88.46%
NRDD→T2	2		21349558	17224696	86.62%

### Differential expression of genes on direct-sowing and drought stress

**Figure 4:**
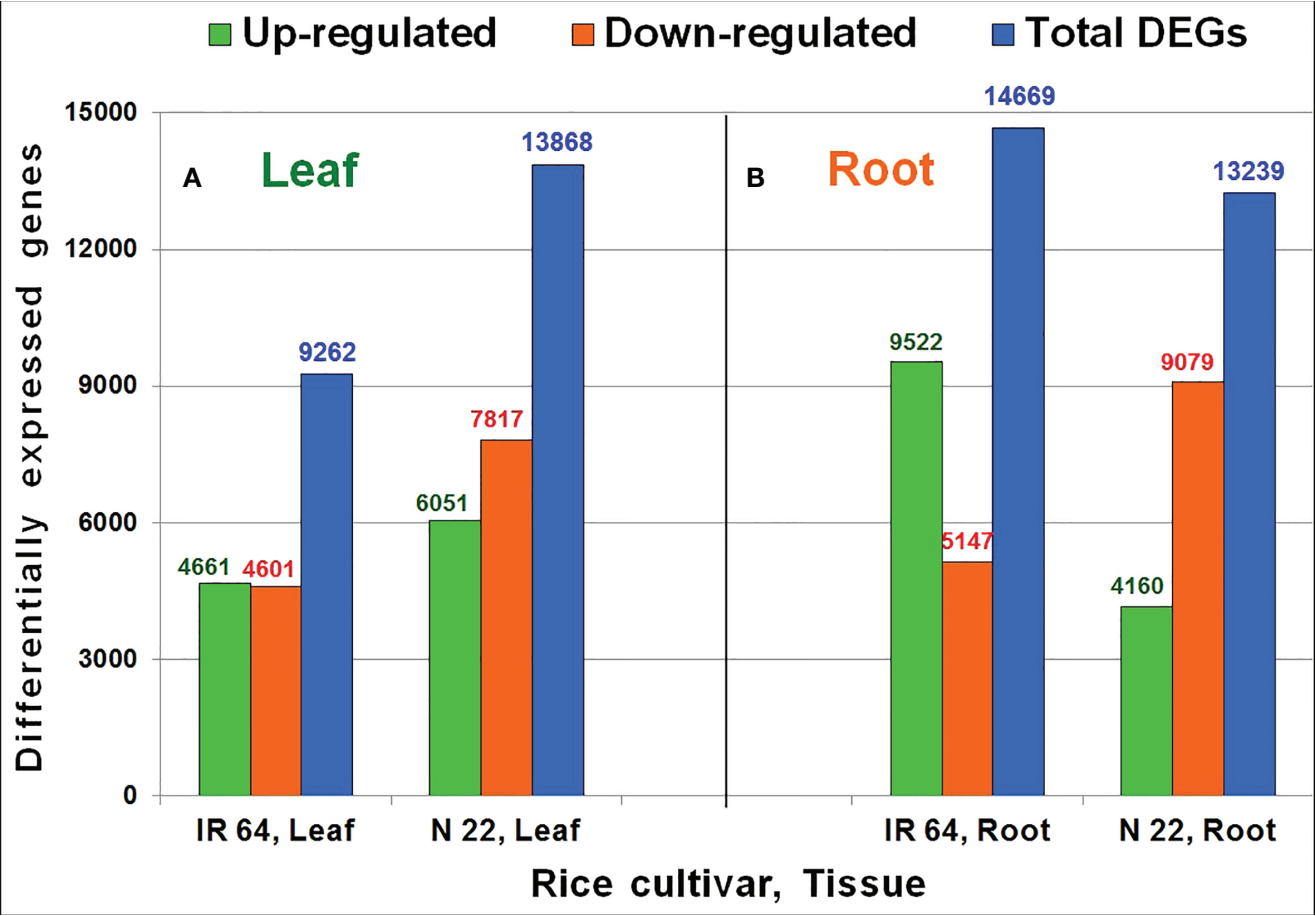
Differentially expressed genes (DEGs) in rice cultivars [IR 64 and Nagina 22 (N 22)] on direct-sowing over transplanting under drought stress imposed at reproductive stage in **(A)** leaf and **(B)** root. Leaf and root tissues were collected at the panicle-initiation stage of the plant for RNA-seq analysis.

Comparative analysis of RNA-seq data from leaf and root tissues grown by different methods of planting and subjected to drought stress was used to identify DEGs under direct-sown conditions over transplanting. In leaf of N 22, about 6051 genes were up-regulated compared to that (4661) in leaf of IR 64 on direct-sowing over transplanting under drought stress. Likewise, a higher number (7817) of genes were down-regulated in leaf of N 22 compared to 4601 genes down-regulated in leaf of IR 64 on direct-sowing over transplanting under the stress (**Figure 4A**). However, in root of N 22 grown by direct-sowing, only 4160 genes were up-regulated compared to a higher number (9522) of genes up-regulated in root of IR 64 on direct-sowing over transplanting under the stress. Moreover, a significantly higher number (9079) of genes were down-regulated in root of N 22 on direct-sowing (over transplanting) under the stress compared to 5147 genes down-regulated in root of IR 64 (**Figure 4B**). Thus, an increase in the total number of DEGs (particularly down-regulated genes) in leaf but a decrease in the total number of DEGs (particularly the up-regulated genes) in root was observed in N 22 compared to that in IR 64 on direct-sowing over transplanting under drought stress (**Figure 4**).

**Figure 5:**
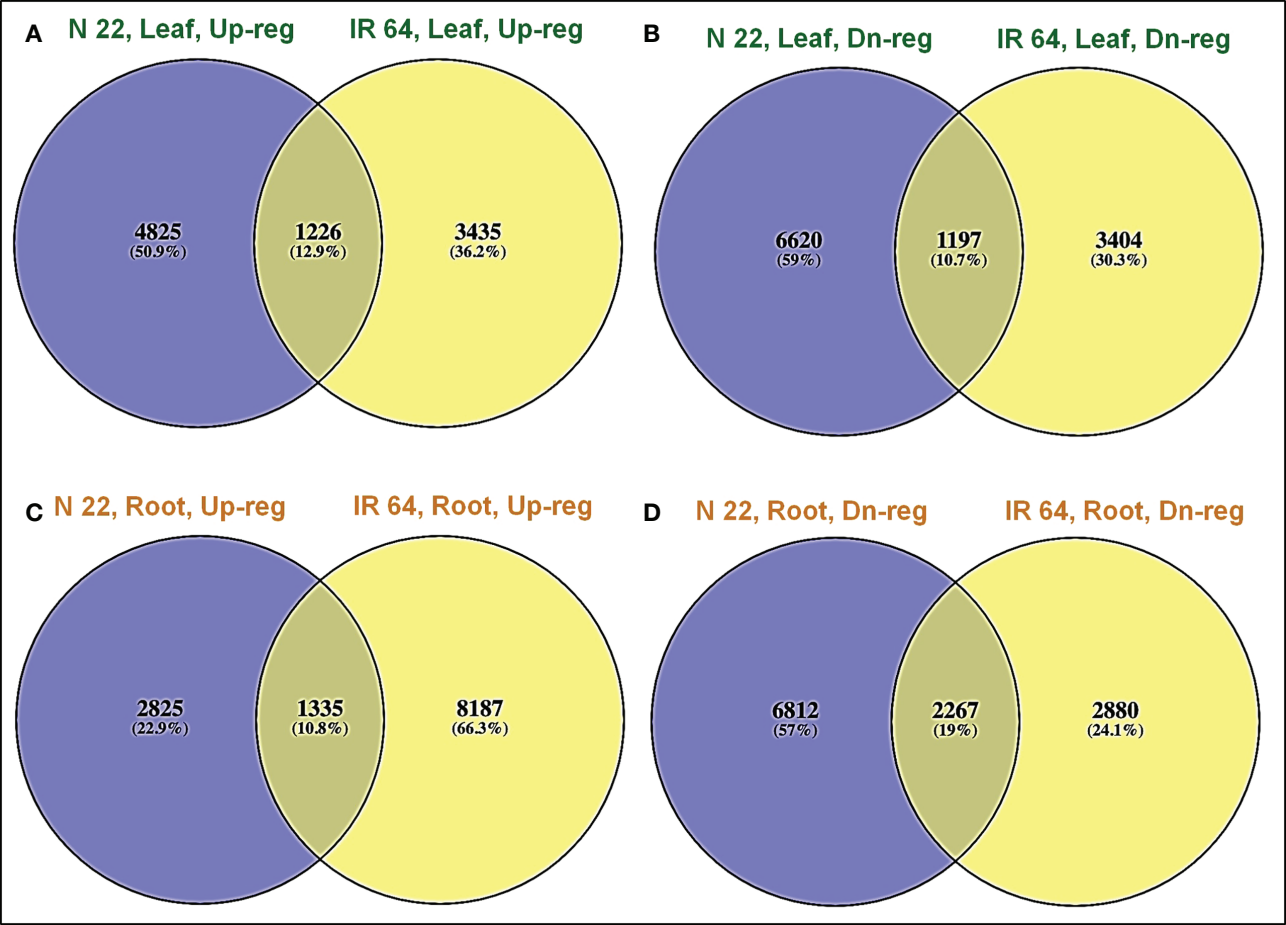
Venn diagram showing differential expression of genes in rice cultivars [IR 64 and Nagina 22 (N 22)] on direct-sowing over transplanting under drought stress. **(A)** Up-regulated, **(B)** down-regulated genes in leaf, **(C)** up-regulated, **(D)** down-regulated genes in root of N 22 and IR 64.

A significantly higher number (4825) of genes were up-regulated exclusively in leaf of N 22 on direct-sowing compared to that (3435) in the leaf of IR 64 (**Figure 5A**). More importantly, 6620 genes were down-regulated exclusively in leaf of N 22 which was considerably lesser than that (3404) in the leaf of IR 64 (**Figure 5B**). A lesser number (2825) of genes were up-regulated exclusively in root of N 22 compared to that (8187) in root of IR 64 (**Figure 5C**). However, a considerably higher number (6812) of genes were down-regulated exclusively in root of N 22 under direct-sown conditions compared to 2880 genes down-regulated in root of IR 64 (**Figure 5D**).

### Highly up-regulated genes in leaf under direct-sown and drought conditions

A large number of genes were observed to be considerably up-regulated (>9-fold) in the leaf of N 22 under direct-sown and reproductive stage drought stress conditions (**Supplementary Table S2**). LOC_Os09g17560 and LOC_Os12g12090 were up-regulated exclusively in N 22 under direct-sown and drought conditions compared to that in IR 64. A large number of (~141) genes (e.g. LOC_Os03g61160, LOC_Os06g28050, LOC_Os06g46740, LOC_Os06g21210, LOC_Os06g28194 etc.) were observed to be considerably (>9-fold) up-regulated in leaf of N 22 under direct-sown drought conditions. Only eight genes (LOC_Os04g33920, LOC_Os11g43790, LOC_Os09g36680, LOC_Os06g51440, LOC_Os06g46740, LOC_Os05g49100, LOC_Os03g21260, LOC_Os03g61160) were observed to be >9-fold up-regulated in leaf of IR 64 under direct-sown and drought conditions. Under transplanted and drought stress conditions, a different set of genes (LOC_Os01g62600, LOC_Os11g26790, LOC_Os08g34390, LOC_Os11g43790, LOC_Os09g36680) were observed to be >9-fold up-regulated, while LOC_Os05g49100 and LOC_Os03g21260 (the genes considerably up-regulated under the direct-sown conditions) were less up-regulated under transplanted conditions in leaf of IR 64 (**Supplementary Table S2**).

### Highly down-regulated genes on direct-sowing and drought stress

A larger number of (197) genes were observed to be considerably (>12-fold) down-regulated in leaf of N 22 under direct-sown and drought conditions (**Supplementary Table S3**). These genes were either less down-regulated or up-regulated in leaf of IR 64 under direct-sown and drought conditions. More importantly, these genes were either less (<7.5-fold) down-regulated or not affected (NA) in leaf of N 22 under transplanted and drought conditions. Furthermore, some of the genes like zinc-binding protein (LOC_Os01g33350), basic Helix-Loop-Helix (LOC_Os02g34320), Gamma-thionin (LOC_Os02g07624), Histone-like transcription factor (LOC_Os02g49410), an expressed protein (LOC_Os08g04740), and a retrotransposon (LOC_Os09g23980) were observed to be down-regulated exclusively in leaf of N 22 under direct-sown and drought conditions. Thirteen genes including POEI4 (LOC_Os10g05790), FBD (LOC_Os01g41370), zinc finger (LOCs_Os01g07930), gibberellin 20 oxidase (LOC_Os07g07420), trehalose-6-phosphate synthase (LOC_Os09g25890), carboxyl-terminal proteinase (LOC_Os02g54960), zinc-binding protein (LOC_Os01g33370), and plastocyanin (LOC_Os02g49350) were observed to be down-regulated exclusively in N 22 under direct-sown and drought conditions (**Supplementary Table S3**).

In root, a significantly higher number (218) of genes were observed to be >8-fold down-regulated in N 22 under direct-sown and drought stress conditions (**Supplementary Table S4**). These genes were either less down-regulated or up-regulated in root of IR 64 under direct-sown and drought conditions. Most of these genes were either less down-regulated or not affected (NA) in root of N 22 under transplanted and drought stress conditions. More importantly, genes for seven proteins viz. LTPL145 - Protease inhibitor (LOC_Os10g40520), thiol protease SEN102 precursor (LOC_Os09g39160), glycosyl hydrolase (LOC_Os11g47550), Peroxidase precursor (LOC_Os03g25300), expressed proteins (LOC_Os01g55060, LOC_Os07g45460), and a dirigent (LOC_Os10g18760) were observed to be down-regulated exclusively in root of N 22 under direct-sown and drought conditions. Four genes coding for the proteins namely LTPL121-protease inhibitor (LOC_Os04g46820), AP2 domain-containing protein (LOC_Os04g46410), Peroxidase precursor (LOC_Os12g34524), and MYB family transcription factor (LOC_Os01g36460) were observed to be down-regulated exclusively in root as well as leaf of N 22 under direct-sown and drought conditions (**Supplementary Table S4**).

### Pathway enrichment gene ontology analysis on direct-sowing and drought stress

**Figure 6:**
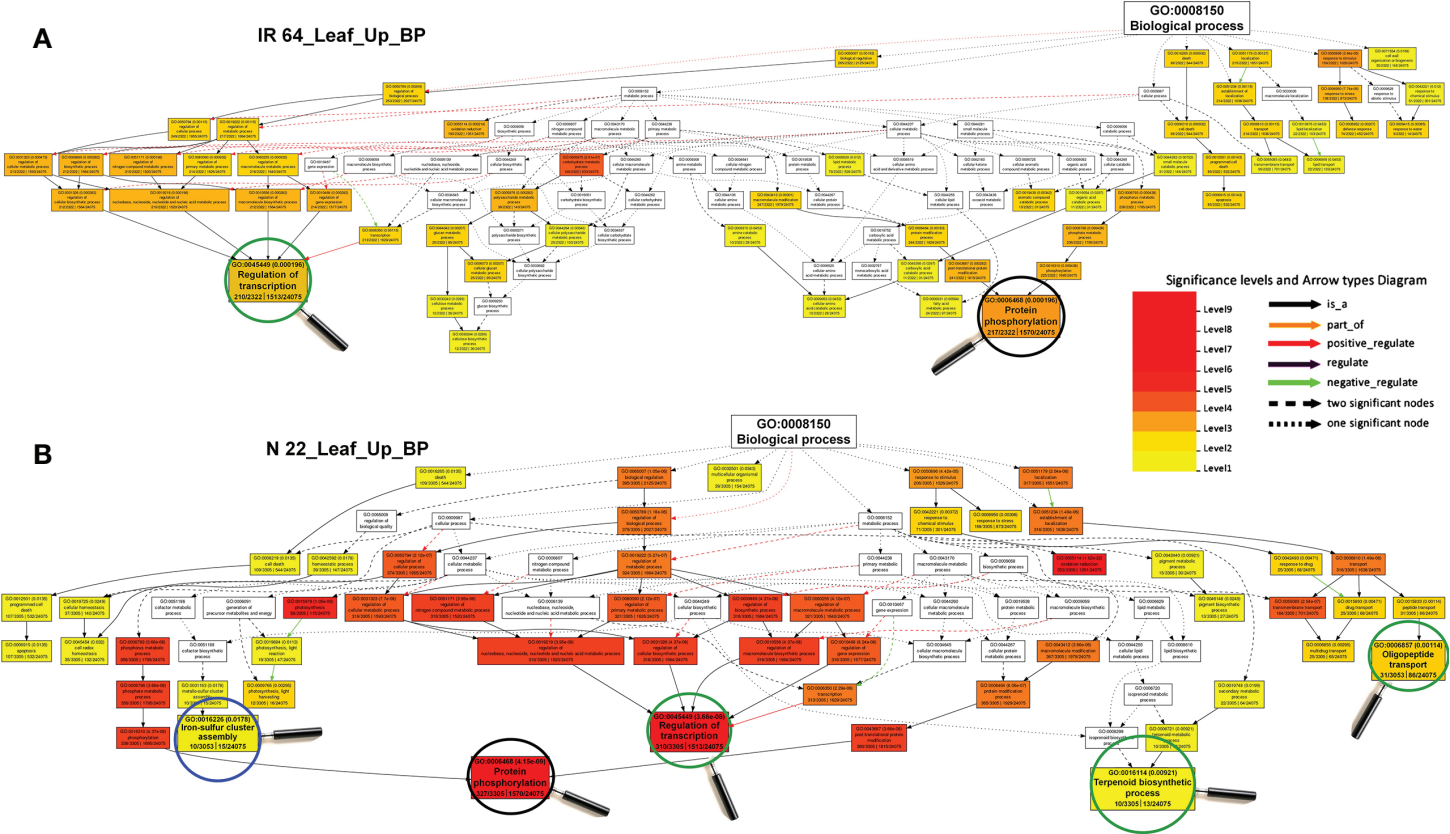
Gene ontology (GO) analysis of enriched biological processes under direct-sown over transplanted conditions in leaf of rice cultivars under drought stress. **(A)** Over-represented GO terms in the leaf of IR 64, and **(B)** over-represented GO terms in the leaf of Nagina 22 rice cultivar.

GO analysis of the terms associated with genetic plasticity of rice to DSR conditions, especially under drought stress, revealed certain biological processes (BP), including regulation of transcription and protein phosphorylation to be comparatively more enriched, while iron-sulfur cluster assembly, terpenoid biosynthetic process, and oligopeptide transport were over-represented exclusively in leaf of N 22, compared to that in the leaf of IR 64 (**Figure 6**). However, BP terms like initiation of DNA replication, glucose metabolic process, glucan biosynthetic process, etc. were under-represented in leaf of N 22, while protein phosphorylation was under-represented in the leaf of IR 64 (**Supplementary Figure S3**). Analysis of the GO terms for BP in root indicated tryptophan and chitin metabolic processes to be significantly enriched in N 22, while RNA-dependent DNA replication and chromatin assembly/disassembly to be over-presented in root of IR 64 under drought stress grown by direct-sowing (**Supplementary Figure S4**). BP terms for regulation of transcription, RNA-dependent DNA replication, nucleosome assembly, cellular glucan metabolic process, and lignin catabolic process were under-represented in root of N 22, while amino acid catabolic process, cellulose biosynthetic process, and protein ubiquitination were under-represented in root of IR 64 (**Supplementary Figure S5**).

**Figure 7:**
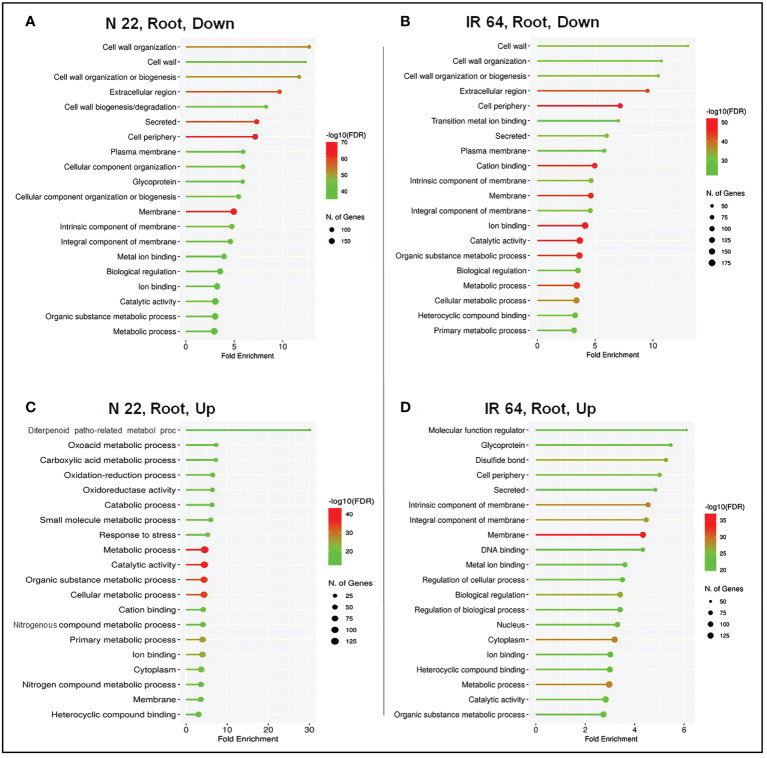
Pathway enrichment analysis on direct-sowing imposed with drought stress in root of rice cultivars. **(A)** Under-represented process/pathway in the root of N 22, **(B)** under-represented process/pathway in IR 64 root, **(C)** over-represented process/pathway in N 22 root, and **(D)** over-represented process/pathway in the root of IR 64. Y-axis shows the name of the process/pathway, and the X-axis shows fold enrichment. Dot-size represents the number of genes and the color indicates the FDR value.

Pathway enrichment analysis in root under DSR conditions for acclimatization of rice was analysed by GO enrichment analysis. The top 20 biological processes significantly (*P* < 0.05) enriched under drought stress were considered for comparative analysis in N 22 and IR 64 cultivars. In root of N 22, the under-represented GO terms included the ‘cellular component organisation’, ‘glycoprotein’, ‘metal ion binding’, etc. (**Figure 7A**), whereas, in root of IR 64 ‘transition metal ion binding’, ‘cation binding’, ‘primary metabolic process’, etc. were the under-represented GO terms (**Figure 7B**). Moreover, the highly enriched GO terms in root of N 22 included the ‘diterpenoid pathogenesis-related metabolic process’, ‘oxidation-reduction process’, ‘response to stress’, ‘nitrogenous compound metabolic process’ etc. (**Figure 7C**), whereas the enriched GO terms in root of IR 64 included the ‘glycoprotein’, ‘disulfide bond’, ‘DNA binding’, etc. (**Figure 7D**).

The pathway enrichment analysis in leaf of N 22 under DSR conditions with drought stress indicated under-represented GO terms for cellular component organisation, DNA binding, regulation of macromolecule metabolic process, catalytic activity, ion binding, etc. (**Supplementary Figure 6A**), whereas, in leaf of IR 64 ion transport, integral component of membrane, cation binding, cellular metabolic process, etc. were comparatively more under-represented (**Supplementary Figure 6B**). Similarly, in leaf of N 22 the GO terms chloroplast, thylakoid, transit peptide, oxidoreductase activity, metal ion binding, etc. were highly enriched (**Supplementary Figure 6C**), whereas, carbohydrate metabolic process, glycoprotein, plasma membrane, cation binding, etc. were comparatively less enriched in leaf of IR 64 (**Supplementary Figure 6D**).

### Differential expression of genes for stress responses

Comparative analysis of the genes for responses to stress under direct-sowing and drought conditions revealed that more than 53 genes were >2-fold up-regulated in leaf of N 22. Only 10 of these were highly (>5-fold) up-regulated in leaf and root of IR 64 under direct-sowing and drought conditions. However, only six of these genes were >5-fold up-regulated in root of N 22 under direct-sowing as well as transplanted conditions imposed with drought stress (**Supplementary Table S5**). The most highly up-regulated genes in leaf of N 22 under direct-sown and drought stress conditions, compared to that in root as well as transplanted conditions, include those for dehydrin proteins (LOC_Os11g26790, LOC_Os11g26780, LOC_Os11g26780, LOC_Os11g26760, LOC_Os11g26750, LOC_Os11g26570), Universal stress protein domain-containing proteins (LOC_Os05g28740, LOC_Os12g36640, LOC_Os02g47840, LOC_Os05g07810, LOC_Os01g32780, LOC_Os01g57450, LOC_Os05g06500), Peroxidase precursor (LOC_Os07g48010LOC_Os07g48020, LOC_Os07g48040, LOC_Os07g47990, LOC_Os07g48050, LOC_Os07g48060), abscisic stress-ripening proteins (LOC_Os01g73250, LOC_Os11g06720, LOC_Os04g34600, LOC_Os01g72900), etc. (**Supplementary Table S5**).

### Differential expression of genes for redox homeostasis

A similar comparative analysis of the genes for redox homeostasis under direct-sowing and drought conditions revealed that more than 26 genes were >2-fold up-regulated in leaf of N 22. Only 3 of these were just 2-fold up-regulated in root of N 22 under direct-sowing and drought conditions, while a few of them were observed to be up-regulated in leaf and root of IR 64 under direct-sowing and drought conditions (**Supplementary Table S6**). However, only a few genes were up-regulated in leaf and root of N 22 as well as IR 64 under transplanted conditions imposed with drought stress. The most highly up-regulated genes in leaf of N 22 under direct-sown and drought conditions, compared to that in root as well as under transplanted conditions, include those for thioredoxins (11 genes), glutaredoxins (11 genes), peroxiredoxins (2 genes), dehydration-responsive element-binding protein (LOC_Os04g36640), etc. (**Supplementary Table S6**).

### Differential expression of transcription factors on direct-sowing and drought stress

More than eight different families of transcription factors (TFs) were observed to be considerably (>11−2-fold) up-regulated in leaf of N 22 on direct-sowing and drought stress. Thirty-five WRKY family, thirty-three MYB family, 33 ‘No apical meristem’ family, 19 helix-loop-helix family, 17 AP2 family, 13 bZIP family, 22 homeodomain-containing TFs, 6 ethylene-responsive TFs, etc. were observed to be more than 2-fold up-regulated in the leaf of N 22 under direct-sown and drought stress conditions (**Supplementary Table S7**). However, the expression of only some of these TFs (e.g. LOC_Os07g27670, LOC_Os02g49986, LOC_Os06g04090, LOC_Os09g28210, LOC_Os03g56010, LOC_Os05g49700) were observed to be up-regulated in leaf of IR 64 under direct-sown and drought conditions. Moreover, TFs of nine other families (including ethylene- and dehydration-responsive TFs, HSF, NAC domain, and MAD box TFs) were also highly up-regulated in leaf of N 22 under direct-sown and drought conditions. Only a few TFs like dehydration-responsive TF (LOC_Os10g38000), HSF (LOC_Os01g39020, LOC_Os06g35960), and MADS74 (LOC_Os12g21880) were more up-regulated in leaf of IR 64 under direct-sown and drought conditions (**Supplementary Table S7**). Some of the TFs like WRKY54 (LOC_Os05g40080), MYB (LOC_Os06g02250), Helix-loop-helix DNA-binding protein (LOC_Os05g51820, LOC_Os05g46370), AP2 (LOC_Os03g22170), HSF (LOC_Os06g36930), and MADS-box family (LOC_Os05g23780) were up-regulated exclusively in leaf of N 22 under direct-sown and drought conditions (**Supplementary Table S7**).

### Differential expression of genes involved in two-component signaling

The genes involved in two-component signaling under direct-sowing and drought conditions revealed that more than 16 genes were >2-fold up-regulated in root of N 22. Only 3 of these were found to be >2-fold up-regulated in root of N 22 under direct-sown and drought stress conditions. None of these genes was >1.5-fold up-regulated in leaf of IR 64 under direct-sown and drought conditions (**Supplementary Table S8**). The highly (>4.0-fold) up-regulated genes in leaf of N 22 under direct-sown and drought conditions, compared to that in root as well as transplanted and drought conditions, including those for OsRR4 type-A response regulator (LOC_Os01g72330), histidine-containing phosphotransfer protein (LOC_Os05g44570), two-component response regulators (LOC_Os02g55320, LOC_Os02g08500), histidine kinases (LOC_Os01g69920, LOC_Os10g21810), OsRR1 type-A response regulator (LOC_Os04g36070), etc. (**Supplementary Table S8**).

### Differential expression of genes for photosynthetic process

More than 16 genes associated with the photosynthesis process (e.g. LOC_Os09g17740, LOC_Os04g16770, LOC_Os01g71700, LOC_Os10g41689) were observed to be >2-fold up-regulated in the leaf of N 22 under direct-sown and drought stress conditions (**Supplementary Table S9**). Some of these genes including chlorophyll A-B binding proteins, photosynthetic reaction center protein, oxygen-evolving enhancer protein 1, cytochrome b6-f complex subunit 4, Amino acid permease family protein, photosystem II D2 proteins, etc. were up-regulated in the leaf of N 22, compared to that in root of N 22 and leaf of IR 64, under direct-sown and drought conditions. However, several of these genes were down-regulated in leaf of IR 64 under drought stress in both direct-sown as well as transplanted conditions (**Supplementary Table S9**).

### Differential expression of genes for carbohydrate metabolic processes

**Figure 8:**
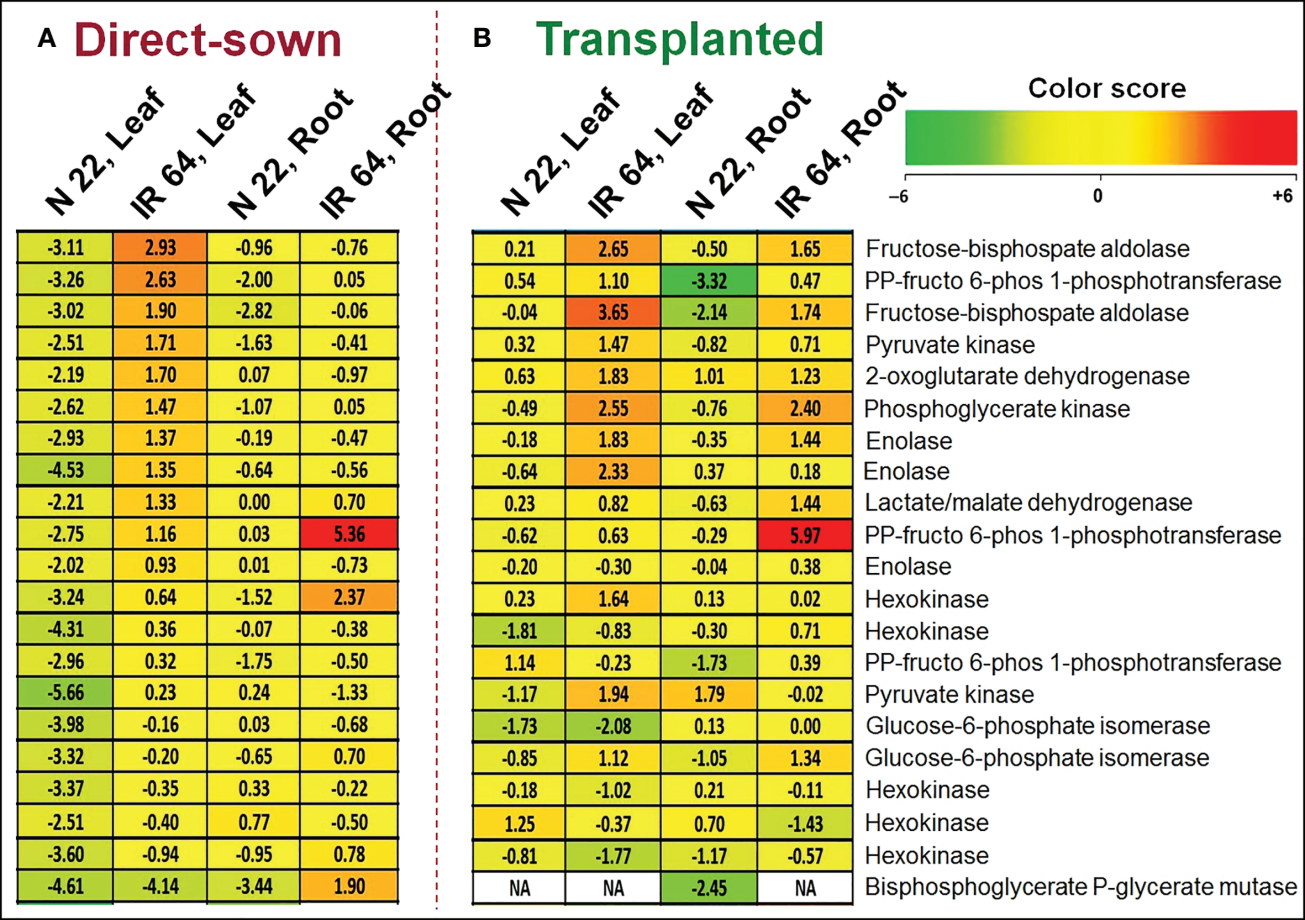
Differential expression of genes for carbohydrate metabolic processes. **(A)** Differentially expressed genes (DEGs) in leaf and root of Nagina 22 and IR 64 under direct-sown conditions subjected to drought stress at the reproductive stage of plant growth, **(B)** DEGs in leaf and root of the rice cultivars under transplanted conditions subjected to drought stress. The expression level of the genes is represented at log2 Fold Change under drought stress over the control.

Many of the genes involved in carbohydrate metabolism/glucose catabolism were up-regulated in leaf of IR 64 under direct-sown and drought stress, compared to their expression under control conditions in root. However, these genes were significantly down-regulated in leaf of N 22 under direct-sown and drought stress conditions (**Figure 8**). Some of these genes were up-regulated in root of IR 64 but remained down-regulated in root of N 22, under direct-sown and drought stress conditions. More importantly, many of these genes were up-regulated in leaf and root of IR 64 as well as N 22 under transplanted and drought stress conditions. The gene for the rate-limiting enzyme of the glycolysis pathway (Phosphofructokinase, LOC_Os09g30240) was observed to be highly (>9-fold) down-regulated in the leaf of N 22 under direct-sown and drought conditions. Moreover, the gene for fructose-bisphosphate aldolase (LOC_Os10g08022) was recorded to be considerably (~11-fold) down-regulated in leaf of N 22 under direct-sown and drought stress conditions. This gene is involved in several vital processes including glycolysis, gluconeogenesis, Calvin cycle and plays a significant role in biotic and abiotic stress responses, regulating the growth and development of plants (**Supplementary Table S10**).

### Differential Expression of genes for calcium-binding proteins

Differential expression of 30 genes coding for calcium-binding proteins was observed to be up-regulated in the leaf of N 22 under direct-sown and drought stress conditions (**Supplementary Table S11**), while no significant up-regulation was observed in root of N 22. The most up-regulated genes included those for OsWAK receptor-like protein kinases (8 genes), calmodulin-related calcium sensor proteins (7 genes), annexin (2 genes), calreticulin precursor proteins (2 genes), etc. Only some of these genes were up-regulated in leaf and root of the rice cultivars under transplanted and drought conditions (**Supplementary Table S11**).

### Differential expression of genes for transmembrane transport

More than 36 genes for transmembrane transporters were significantly up-regulated in leaf of N 22 under direct-sown, drought conditions (**Supplementary Table S12**). Some of the up-regulated genes include sodium/calcium exchanger proteins, MATE efflux family proteins, inorganic phosphate transporters, auxin-efflux carrier components, ammonium transporter proteins, citrate transporters, potassium channels, sulfate transporters, etc. Only some of these genes were up-regulated in root of N 22 and leaf/root of IR 64 under direct-sown, drought stress conditions (**Supplementary Table S12**).

### Differential expression of genes for terpenoid biosynthesis

More than 10 genes coding for the enzymes involved in terpenoid biosynthesis were up-regulated in leaf of N 22 under direct-sown, drought stress conditions by >2-fold (**Supplementary Table S13**). Most of these genes were down-regulated in leaf of IR 64, while a few of them were up-regulated in root of the rice cultivars under direct-sown, drought-stress conditions. On the contrary, some of these genes were up-regulated in root of the rice cultivars under transplanted, drought stress conditions. Some of the up-regulated genes in leaf of N 22 include zeaxanthin epoxidase-chloroplast precursor, lycopene beta cyclase, transketolase, amine oxidase, etc. (**Supplementary Table S13**).

### Differential expression of genes for chromatin assembly and epigenetic modification

Under direct-sown drought-stressed conditions, more than 7 genes associated with epigenetic modification of DNA, histone proteins, and chromatin/nucleosome assembly were observed to be up-regulated in N 22 under direct-sown, drought stress conditions (**Supplementary Table S14**). The genes up-regulated in leaf of N 22 under direct-sown, drought stress conditions included methyltransferase (LOC_Os03g56370), SNF7 domain-containing protein (LOC_Os05g01250), core histone H2A/H2B/H3/H4 (LOC_Os03g14669), histone-like transcription factor (LOC_Os08g07740), etc. Likewise, the genes up-regulated in root of N 22 under direct-sown and drought stress conditions included those for methyltransferases (LOC_Os04g11970, LOC_Os02g56020, LOC_Os03g02010), histone-lysine N-methyltransferase (LOC_Os03g20430), core histone H2A/H2B/H3/H4 (LOC_Os03g14669), SNF7 domain-containing protein (LOC_Os05g01250), SET domain-containing protein (LOC_Os02g36710), etc. Under transplanted drought-stressed conditions only some of the genes were up-regulated in leaf and root tissue of the rice cultivars (**Supplementary Table S14**).

### Differential expression of genes for stress responses on changing the planting method

When N 22 grown continuously by direct-sowing for five years was subjected to drought stress a large number (53) of genes for stress response showed up-regulated expression in leaf, but on changing the method of planting (i.e. transplanting in the sixth year) and subjected to drought stress the genes for ‘response to stress’ showed reduced/down-regulated expression in leaf of N 22 (**Supplementary Table S15**). Interestingly, when the N 22 seeds collected from the plants continuously grown by transplanting were used for direct-sowing and exposed to drought stress, the genes for stress response were up-regulated in leaf of N 22, as if they resumed their expression level (**Supplementary Table S15**).

### Differential expression of genes for redox homeostasis on changing the planting method

A large number (26) of genes for redox homeostasis showed up-regulated in leaf of N 22 when grown by direct-sowing and drought stress, but on changing the method of planting (i.e. transplanting) and exposure to drought stress the genes for redox homeostasis showed down-regulated expression in leaf of N 22 (**Supplementary Table S16**). When N 22 seeds collected from the plants continuously grown by transplanting were grown by direct-sowing and subjected to drought stress, the genes for redox homeostasis showed up-regulated expression in leaf of N 22 (**Supplementary Table S16**).

### Differential expression of genes for transcription factors on changing the method of planting

In leaf of N 22 continuously grown under direct-sown conditions for several generations and subjected to drought stress showed up-regulated expression of a large number (186) of genes for transcription factors (**Supplementary Table S17**), but on changing the method of planting i.e. transplanting and subjecting to drought stress the genes for transcription factors demonstrated reduced or down-regulated expression in leaf. Remarkably, when N 22 seeds collected from the plants continuously grown by transplanting were grown by direct-sowing and exposed to drought stress, the genes for transcription factors resumed their up-regulated expression in leaf (**Supplementary Table S17**).

### Differential expression of genes for epigenetic regulation of gene expression on changing method of planting

A large number (13) of genes involved in epigenetic regulation of gene expression showed up-regulated expression in leaf of N 22 continuously grown by direct-sowing and subjected to drought stress. Such genes included those for chromatin assembly and epigenetic regulation of gene expression (**Supplementary Table S18**). Interestingly, when the seeds of N 22 collected from the plants continuously grown by transplanting were grown by changing the method of planting, i.e. direct-sowing, and subjected to drought stress, the genes for chromatin assembly/epigenetic regulation resumed their up-regulated expression in leaf (**Supplementary Table S18**).

### Validation of RNA-seq data by RT-qPCR analysis

**Figure 9:**
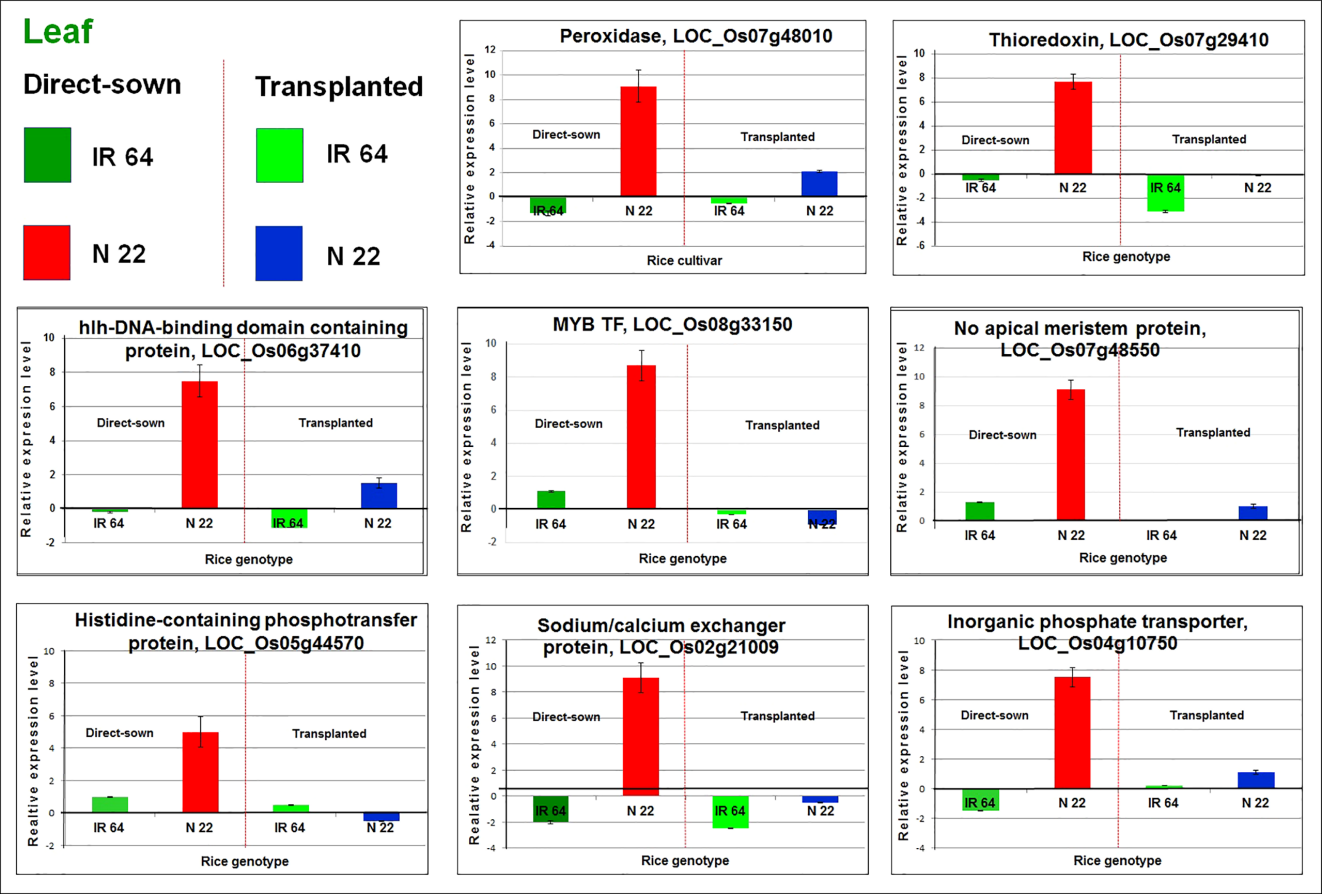
RT-qPCR validation of eight randomly selected differentially expressed genes in leaf of Nagina 22 (N 22). The cDNA was prepared from total RNA isolated from leaf of N 22 and IR 64 grown by transplanting and direct-sowing subjected to drought stress at the reproductive stage. Data represent the mean ± SD (n = 3).

The expression profile of eight randomly selected differentially expressed genes (as recorded by the RNA-sequencing) in the rice cultivars on direct-sowing and transplanting was validated using Reverse Transcriptase quantitative Polymerase Chain Reaction (RT-qPCR). The RT-qPCR analysis demonstrated an agreement between the expression pattern of the genes by RNA-seq and RT-qPCR analyses (**Figure 9**). Thus, the trustworthiness of the RNA-seq data was confirmed.

## Discussion

Dry/direct-sown rice (DSR) is emerging as a resource-conserving and climate-smart alternative to TPR (Shekhawat et al., 2020). However, no specific variety has been bred for dry/direct-sown conditions. The effects of planting methods on germination/seedling-vigor, growth and development of plant indicated role of germination-vigor on the success of rice crops, particularly those grown by dry/direct-sowing (Kumar et al., 2022a). To unravel the genes/pathways responsible for the genetic plasticity of rice to different methods of planting under drought stress (at the reproductive stage), which is a common and frequent abiotic stress occurring on dry/direct-sowing of rice, we performed a comparative RNA-seq analysis of two *indica* rice cultivars.

A significant difference in root morphology of the rice cultivars at the seedling stage grown by direct-sowing affirms the importance of germination vigor, root architecture, and stand establishment in better performance of the cultivar selected for this study. The horizontal spread of roots of IR 64, while vertical growth of roots of N 22 seedlings on direct-sowing must be helpful in the better establishment of N 22 seedlings even at lower moisture content under DSR conditions (**Figure 1A**). However, equally good root architecture of N 22 and IR 64 seedlings raised in the nursery (**Supplementary Figure S2**) but the shallow root system of IR 64 on dry/direct-sowing corroborated with the reason behind the poor performance of IR 64 under DSR conditions (Seem and Kumar, 2021). Lower soil moisture under DSR conditions significantly affects the germination of IR 64 seeds compared to that in the case of N 22 (Kumar, 2021). Reduced SMC (about one-third) at the reproductive stage of growth under DSR conditions, compared to that under transplanted conditions, caused significantly lower RWC in leaf of IR 64 than that in the leaf of N 22, which was further reduced under drought stress. Better root architecture and efficient water management by leaves of N 22 are responsible for the better performance of N 22 under DSR and drought stress conditions compared to that of IR 64. DSR faces repeated drought stress, of course of varying intensity, throughout its presence in the field. In contrast, TPR is continuously irrigated to maintain humidity in the field. Reproductive stage drought has been reported to cause adverse effects on the productivity of rice (Vikram et al., 2011; Liu et al., 2014; Palanog et al., 2014). The role of the root system architecture (RSA) in the better performance of N 22 under DSR and drought stress conditions (**Figure 1B**) corroborates with the findings reported earlier (Sandhu et al., 2019).

Agronomic performance of N 22 under DSR conditions, in terms of the number of tillers, panicles, grains, and test weight, was comparable with that under TPR conditions while it was significantly lower in the case of IR 64 (**Figure 2**). Moreover, the performance of the rice cultivars in terms of protein yield also indicated the equally good performance of N 22 under DSR conditions, while increased protein content in the seeds (**Figure 3**) with reduced grain yield of IR 64 demonstrated the deleterious effects of stresses the plants might have experienced under DSR conditions.

An average of 23 million reads for each pooled RNA sample sequenced in duplicates with higher mapping efficiency (**Table 1**) indicate sufficiently good quality of the data generated. A higher number of differentially expressed genes (13868) in leaf of N 22 with 6051 genes up-regulated and 7817 genes down-regulated, compared to about 4600 genes up- as well as down-regulated in leaf of IR 64, on direct-sowing (over transplanting), indicated important roles of leaf in providing genetic plasticity to N 22, particularly through down-regulation of the genes (**Figure 4A**). About 4000 more genes down-regulated in root of N 22 might be responsible for the better performance of this cultivar under DSR and drought stress conditions (**Figure 4B**). Many of these genes are involved directly or indirectly in mitigating the wastage/excessive use of water. While about one hundred additional genes down-regulated in root of N 22, compared to those down-regulated in root of IR 64, under DSR conditions (without severe drought stress) (**Supplementary Figure S7**) might be involved in better performance of N 22 under DSR conditions, about 2600 additional genes gets down-regulated when drought stress was imposed (**Figure 4B**). Exposure of plants to drought stress causes a decrease in the number of up-regulated genes while an increase in the down-regulated genes in leaf of N 22 when grown by direct-sowing (**Figure 4B**, **Supplementary Figure S7**).

The up-regulated expression of 9522 (more than double) genes, with only 5147 (about half) genes, in root of IR 64 under drought stress might be collectively responsible for poor performance of IR 64 under DSR conditions. Moreover, a significantly higher number (4825) of genes up-regulated on direct-sowing exclusively in leaf of N 22, compared to that (3435) in IR 64 (**Figure 5A**), and 6620 genes down-regulated exclusively in leaf of N 22 [compared to a lesser (3404) number of genes in IR 64] (**Figure 5B**) might be responsible for poor performance of IR 64 under DSR conditions. In root of N 22, the exclusively down-regulated genes (6812) under direct-sown conditions and drought stress imposition must be the major players responsible for better performance of N 22 (**Figure 5D**).

The genes up-regulated in leaf of N 22 under direct-sown and reproductive stage drought stress include those for WRKY and MYB TF families, isocitrate lyase, glycine-rich proteins, cupin domain-containing protein, hydrolase, terpene synthase, etc. (**Supplementary Table S2**). These have been reported to play role in various abiotic stress tolerance, cellular stress responses and signaling (Czolpinska and Rurek, 2018; Yuenyong et al., 2019). A methyltransferase up-regulated exclusively in N 22 under direct-sown and drought conditions must be involved in epigenetic modifications in modulating gene expression. Moreover, a larger number of genes down-regulated in leaf of N 22 under direct-sown and drought conditions (**Supplementary Table S3**) including the genes for no apical meristem proteins, glutelins, sucrose and starch synthases, etc. must be involved in saving energy for survival, growth, and productivity of plants under the stress. However, genes for zinc-binding protein, basic Helix-Loop-Helix, gamma-thionin, histone-like transcription factor, and a retrotransposon protein down-regulated exclusively in leaf of N 22 under direct-sown and drought conditions (**Supplementary Table S3**) must be responsible for better performance of N 22 under DSR conditions.

Several genes were down-regulated in root of N 22 under direct-sown and drought stress conditions (**Supplementary Table S4**). The genes for LTPL145 - Protease inhibitor, thiol protease SEN102 precursor, glycosyl hydrolase, peroxidase precursor, and a dirigent protein, down-regulated (>8-fold) exclusively in root of N 22 under direct-sown and drought conditions, must be responsible for protecting it under stressful conditions (**Supplementary Table S4**). Genes for LTPL121-protease inhibitor, AP2 domain containing protein, peroxidase precursor, and MYB family TF, down-regulated exclusively in root and leaf of N 22 under direct-sown and drought conditions, must help manage survival and growth of N 22 under various stresses faced by the plant.

Over-representation of biological processes like iron-sulfur cluster assembly, terpenoid biosynthetic process, and oligopeptide transport exclusively in leaf of N 22 in addition to enrichment of transcription processes and protein phosphorylation, compared to that in the leaf of IR 64 (**Figure 6**), indicate their roles in rendering genotypic plasticity to changing environmental conditions. Moreover, the under-representation of GO terms like initiation of DNA replication, glucose metabolic process, glucan biosynthesis etc. in leaf of N 22, while protein phosphorylation in the leaf of IR 64, (**Supplementary Figure S3**) might help conserve the energy under unfavorable conditions. Likewise, the GO terms like tryptophan and chitin metabolic processes are over-presented in root of N 22, while RNA-dependent DNA replication and chromatin assembly/disassembly in IR 64, under drought stress on direct-sowing (**Supplementary Figure S4**) might be important for protecting the N 22 plant under stress by modulating the gene expression level. Under-representation of the BP terms like regulation of transcription, RNA-dependent DNA replication, nucleosome assembly, cellular glucan metabolic process, and lignin catabolic process in root of N 22, while amino acid catabolic process, cellulose biosynthetic process, and protein ubiquitination in IR 64 (**Supplementary Figure S5**), might help to protect the plant under stress by modulating gene expression level.

Comparative analysis of the genes for stress responses under direct-sowing and drought conditions revealed five times more (53) genes up-regulated in leaf of N 22 compared to that in IR 64. The most highly up-regulated genes in leaf of N 22 under direct-sown, drought stress conditions include those for dehydrin proteins, universal stress protein domain containing proteins, peroxidase precursors, abscisic stress-ripening proteins, etc. (**Supplementary Table S5**) indicating their importance in protecting N 22 plants from stresses. The genes for redox homeostasis including those for thioredoxins, glutaredoxins, peroxiredoxins, dehydration-responsive element-binding protein, etc. were up-regulated in leaf of N 22 under direct-sowing and drought conditions (**Supplementary Table S6**). This indicates the role of redox homeostasis in mitigating the effects of stresses experienced by plants grown under DSR conditions (Hasanuzzaman et al., 2020; Sasi et al., 2021).

Transcription factors of different families, including WRKY, MYB, No apical meristem, helix-loop-helix, AP2, bZIP, homeodomain-containing TFs, ethylene-responsive TFs, etc. were highly up-regulated in the leaf of N 22 compared to that in leaf of IR 64 under DSR and drought stress conditions (**Supplementary Table S7**). Interestingly, some of this TFs like WRKY54, MYB (LOC_Os06g02250), Helix-loop-helix DNA-binding protein (LOC_Os05g51820, LOC_Os05g46370), AP2 (LOC_Os03g22170), HSF (LOC_Os06g36930), and MADS-box family (LOC_Os05g23780) were up-regulated exclusively in leaf of N 22. This might be responsible for modulating the expression of stress-responsive genes under DSR conditions. More interestingly, a large number (16) of genes involved in two-component signaling were up-regulated in leaf of N 22, while only three genes were up-regulated in root of N 22, but not in leaf of IR 64, under direct-sown and drought conditions (**Supplementary Table S8**). This indicates more efficient stress signaling in N 22, wherein root senses the stress (particularly drought stress), and passes on the signals to leaves which efficiently manage the expression of stress-associated genes in N 22.

To our surprise, several of the genes associated with the photosynthetic process (including chlorophyll A-B binding proteins, photosynthetic reaction center protein, oxygen-evolving enhancer protein 1, cytochrome b6-f complex subunit 4, amino acid permease family protein, photosystem II D2 proteins, etc.) were up-regulated in leaf of N 22 under direct-sown and drought stress conditions. Many of these genes were down-regulated in leaf of IR 64 under DSR and drought stress conditions (**Supplementary Table S9**). This indicates that significantly up-regulated expression of 16 genes in leaf of N 22 must be responsible for higher rate of photosynthesis in managing the equally better performance of N 22 under DSR and drought stress conditions. However, the genes involved in carbohydrate metabolism, particularly glucose catabolism, were up-regulated in leaf of IR 64 but significantly down-regulated in leaf of N 22 under direct-sown and drought stress conditions (**Figure 8**). Because of the up-regulated expression of >10 genes involved in carbohydrate metabolic processes, even under abiotic stresses, resulting in a higher metabolic rate under stressful conditions (without an appropriate adjustment) might be responsible for the poor genotypic plasticity of IR 64 to DSR conditions (**Supplementary Table S10**). Interestingly, expression of the rate-limiting enzyme of glycolysis pathway (phosphofructokinase) and fructose-bisphospatealdolase (involved in glycolysis, gluconeogenesis, Calvin cycle, and playing role in biotic/abiotic stress responses, regulating growth and development process) down-regulated in leaf of N 22 under direct-sown and drought conditions might be responsible for the better performance of N 22 (**Supplementary Table S10**).

A considerably large number of genes coding for calcium-binding proteins up-regulated in leaf (compared to that in root) of N 22, while only a few of them up-regulated in IR 64 tissues, under direct-sown and drought stress conditions (**Supplementary Table S11**) indicate the role of calcium signaling in stress management in leaf of N22 (compared to that in IR 64) and adaptation to adverse environmental conditions. Such findings corroborate with those of Wan et al. (2012) and Virdi et al. (2015). Likewise, a large number of genes for transmembrane transporters, up-regulated in leaf of N 22 (but only a few of them up-regulated in root of N 22 or IR 64 tissues) under direct-sown and drought conditions (**Supplementary Table S12**) indicate the importance of efficient water and nutrient transportation in N 22 under DSR/drought conditions as adaptive strategy/response. These observations are in agreement with the earlier findings (Chen et al., 2017; Gill et al., 2021).

Up-regulated expression of the genes involved in terpenoid biosynthesis in leaf of N 22, but down-regulated in leaf of IR 64 under direct-sown and drought stress conditions (**Supplementary Table S13**) indicate better adaptability/genotypic plasticity of N 22, which corroborates with our earlier findings (Kumar et al., 2022a). Terpenoid biosynthesis in plants plays an important ecological role in mediating the interaction between the plant and stress tolerance in protecting from abiotic and biotic stresses (Liu et al., 2018; Zhou et al., 2020). Up-regulated expression of the genes involved in epigenetic modification of DNA, histones, and chromatin/nucleosome assembly in N 22 under direct-sown, drought stress conditions compared to that under transplanted conditions (**Supplementary Table S14**) indicates important roles of epigenetic changes in adaptation of rice to different environmental conditions prevails in the rice field grown by different planting methods. DNA methylation and histone modification are responsible for reprogramming gene expression under changing environmental conditions (Kumar et al., 2022a). Histones being an important component of chromatin structure, stress-induced differential expression of histone-modifying enzymes help modulate gene expression under the stresses (Kumar and Mohapatra, 2021a). DNA methylation, histone modifications, and chromatin remodeling interact with each other towards condensation/decondensation of chromatin, making it accessible or inaccessible to the transcriptional machinery for gene expression facilitating plant growth and development under environmental stresses (Cedar and Bergman, 2009; Kumar et al., 2022b).

The genes for ‘response to stress’ are up-regulated in leaf of N 22 when grown by direct-sowing but they show down-regulated expression when the plants are grown by transplanting. Likewise, the genes for ‘response to stress’ showing lower expression levels in leaf of N 22 on transplanting, show significantly up-regulated expression on growing by direct-sowing (**Supplementary Table S15**). These indicate the involvement of genes in modulating metabolic pathways according to the conditions prevailing under different methods of planting of rice. The genes up-regulated in leaf of N 22 grown by direct-sowing subjected to drought stress showed down-regulated expression in the leaf on growing by transplanting. Moreover, the genes (for redox homeostasis) showing down-regulated expression in leaf of N 22 on transplanting showed up-regulated expression in the leaf when grown by direct-sowing under drought stress (**Supplementary Table S16**). Redox homeostasis is known to play important role in managing environmental stresses in plants (Hasanuzzaman et al., 2020).

Also, 186 genes coding for different transcription factors show up-regulated expression in leaf of N 22 on direct-sowing under drought stress but showed down-regulated expression in the leaf when plants are grown by transplanting (**Supplementary Table S17**). Similarly, the genes involved in epigenetic modifications show up-regulated expression in leaf of N 22 on direct-sowing under drought stress, but the genes show down-regulated expression in the leaf when grown by transplanting. Again, the genes for chromatin assembly/epigenetic regulation showing down-regulated expression in leaf of N 22 on transplanting under the stress appear to be up-regulated in the plants grown by direct-sowing and subjected to drought stress (**Supplementary Table S18**). Such varying expression of a different set of genes in the leaf of N 22 under different planting methods (direct-sowing and transplanting) was not observed in IR 64. This affirms that these genes are involved in the genetic plasticity observed in N 22.

## Conclusions

Dry/direct-sown rice, being an environment-friendly and resource-saving strategy, supports the concept of ‘*producing more from less*’ by saving natural resources (water and soil) and it might also help in mitigating the emission of greenhouse gases. DSR faces several abiotic and biotic stresses in the field, among which drought stress is most common and damaging. Many of the varieties developed for TPR conditions show considerable (~30%) reduction in yield under DSR conditions, which requires identification of the QTLs/genes for DSR-suited traits such as early/uniform germination, seedling vigor, nutrient use-efficiency, etc. This is the first report on decoding the involvement of genes for ‘responses to stress’, redox homeostasis, transcription factors, two-component signaling, photosynthetic process, carbohydrate metabolic processes, calcium-binding proteins, transmembrane transporters, terpenoid biosynthesis, and chromatin assembly/epigenetic modification in fetching genetic plasticity to N 22 for its better adaptation/performance under dry/direct-sown and drought stress conditions. Involvement of the genes in rendering genetic plasticity was confirmed by reversal of the method of planting which showed altered/resumed expression of the genes. Further comprehensive analyses of the mechanisms/pathways involved in genetic plasticity would help improve the adaptability and yield of rice under dry/direct-sown conditions. However, the epigenetic and epitranscriptomic regulation of gene expression [instrumental behind environmental stress tolerance (Kumar et al., 2018; Kumar and Mohapatra, 2021a; Kumar and Mohapatra, 2021b)] need to be understood for possible modulation of genetic plasticity in crop plants. These might help improve rice for dry/direct-sowing, mitigating the effects of global climate change, emission of greenhouse gases for better ecological integrity and efficiency.

## Data availability statement

The data presented in the study are deposited in the NCBI Sequence Read Archive (SRA) database (https://www.ncbi.nlm.nih.gov/sra) repository, accession number PRJNA805549, PRJNA833055, and PRJNA889399.

## Author contributions

TM and SuK conceived the study; SuK performed the studies and data collection. GS and TM supervised the experiments. SaK performed bioinformatics analysis of the data. SuK and SaK prepared the manuscript. SuK, GS and TM revised the manuscript, and all the authors approved the final draft. All authors contributed to the article and approved the submitted version.

## Funding

The research was carried out with financial support from the National Agricultural Science Fund (NASF/ABP-70161/2018-19) from the Indian Council of Agricultural Research, Government of India, New Delhi, India.

## Conflict of interest

Author SaK is employed at the Decode Genomics Private Limited, New Delhi, India.

The remaining authors declare that the research was conducted in the absence of any commercial or financial relationships that could be construed as a potential conflict of interest.

## Publisher’s note

All claims expressed in this article are solely those of the authors and do not necessarily represent those of their affiliated organizations, or those of the publisher, the editors and the reviewers. Any product that may be evaluated in this article, or claim that may be made by its manufacturer, is not guaranteed or endorsed by the publisher.
