# Supplementary material for: Molecular basis of genetic plasticity to varying environmental conditions on growing rice by dry/direct-sowing and exposure to drought stress: Insights for DSR varietal development
Source: Front Plant Sci. 2022 Oct 24;13:1013207. doi: 10.3389/fpls.2022.1013207 (PMC9638133; doi:10.3389/fpls.2022.1013207)
Supplement: Supplementary file 1 [file Table_1.doc]

**Supplementary** **Table S1.** The list of primers used for RT-qPCR validation of some of the randomly selected differentially expressed genes in the contrasting rice (IR 64, Reproductive stage drought sensitive; N 22, Reproductive stage drought tolerant) cultivars.

| **Gene Name** | **Gene ID** | **Forward Primer (5′→ 3′)** | **Reverse Primer (5′→ 3′)** |
| --- | --- | --- | --- |
| Peroxidase | LOC_Os07g48010 | ACCTGCCTCCTCCTTTCTTC | ACCTGAGAGAGCAACCATGT |
| Thioredoxin | LOC_Os07g29410 | TTCCTCTTCATCAGGGACGG | CCTTGACGCCGTTGTATCTG |
| Helix-loop-helix DNA-binding domain containing protein | LOC_Os06g37410 | TTCCTCTTCATCAGGGACGG | CCTTGACGCCGTTGTATCTG |
| MYB family transcription factor | LOC_Os08g33150 | GGATCAACGACAGCTTCCAG | TTGTCCATGTACTCCGTCGT |
| No apical meristem protein | LOC_Os07g48550 | ATTGCGTCTCATTTGCCTGG | CCGTGTTCTTTCTCTCTGCG |
| Histidine-containing phosphotransfer protein | LOC_Os05g44570 | CCTGATGCACAACCACTACG | CTTGCAGAACCTCCGAATCG |
| *OsWAK60* - OsWAK receptor-like protein kinase | LOC_Os04g30240 | AGCAATTGAAAGGCAGCTGT | CCTTCGGCGTTTTCTTCTCC |
| Sodium/calcium exchanger protein | LOC_Os02g21009 | CTTCTTCCTGCTACAACCGC | ATGACCGTGAACTTGTTGGC |
| Inorganic phosphate transporter | LOC_Os04g10750 | GCAGTAATCACCACCGCATT | TGGGAGAGCTTTCATGACGA |
| *Actin* gene | LOC_Os03g50885 | TTGCTGACAGGATGAGCAAG | TGGAATGTGCTGAGAGATGC |
| *β-tubulin* gene | LOC_Os01g59150 | GCTGACCACACCTAGCTTTGG | AGGGAACCTTAGGCAGCATGT |
