# Supplementary material for: Molecular basis of genetic plasticity to varying environmental conditions on growing rice by dry/direct-sowing and exposure to drought stress: Insights for DSR varietal development
Source: Front Plant Sci. 2022 Oct 24;13:1013207. doi: 10.3389/fpls.2022.1013207 (PMC9638133; doi:10.3389/fpls.2022.1013207)

Supplementary Method S1: Experimental design for growing contrasting rice cultivars (Nagina 22, drought tolerant; IR 64, sensitive to reproductive stage drought) by direct-sowing and transplanting for five consecutive years followed by altered method of planting, drought stress imposition by withholding irrigation for 4–5 days (until soil moisture content reduced by 75%) and relative water content of leaves dropped down to ~58%), and collection of leaf and root tissue samples for molecular analysis.

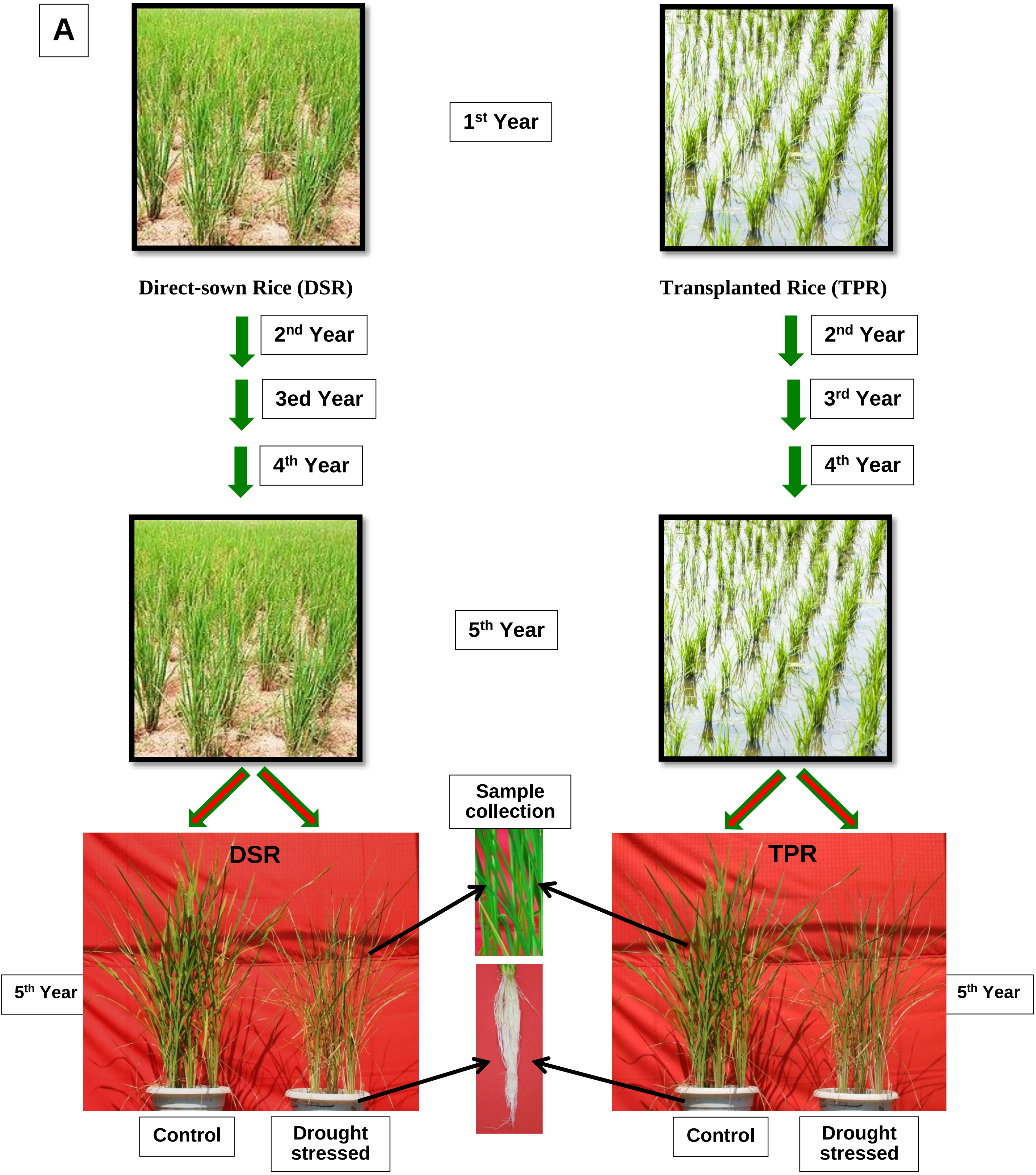

Alteration in method of planting to grow rice (6th year)

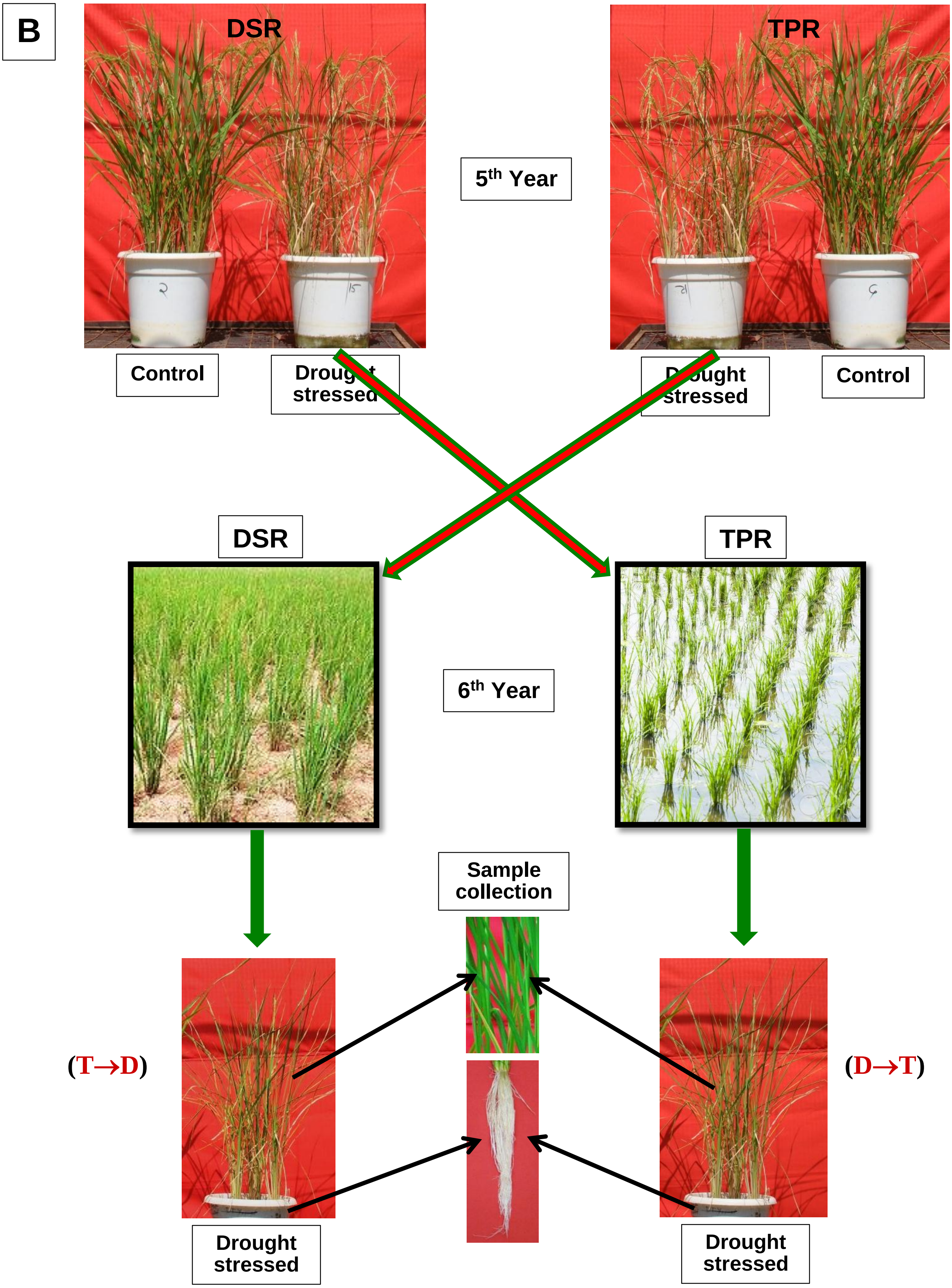

Plant tissue sample collection and RNA isolation

(Biological and Technical replications)

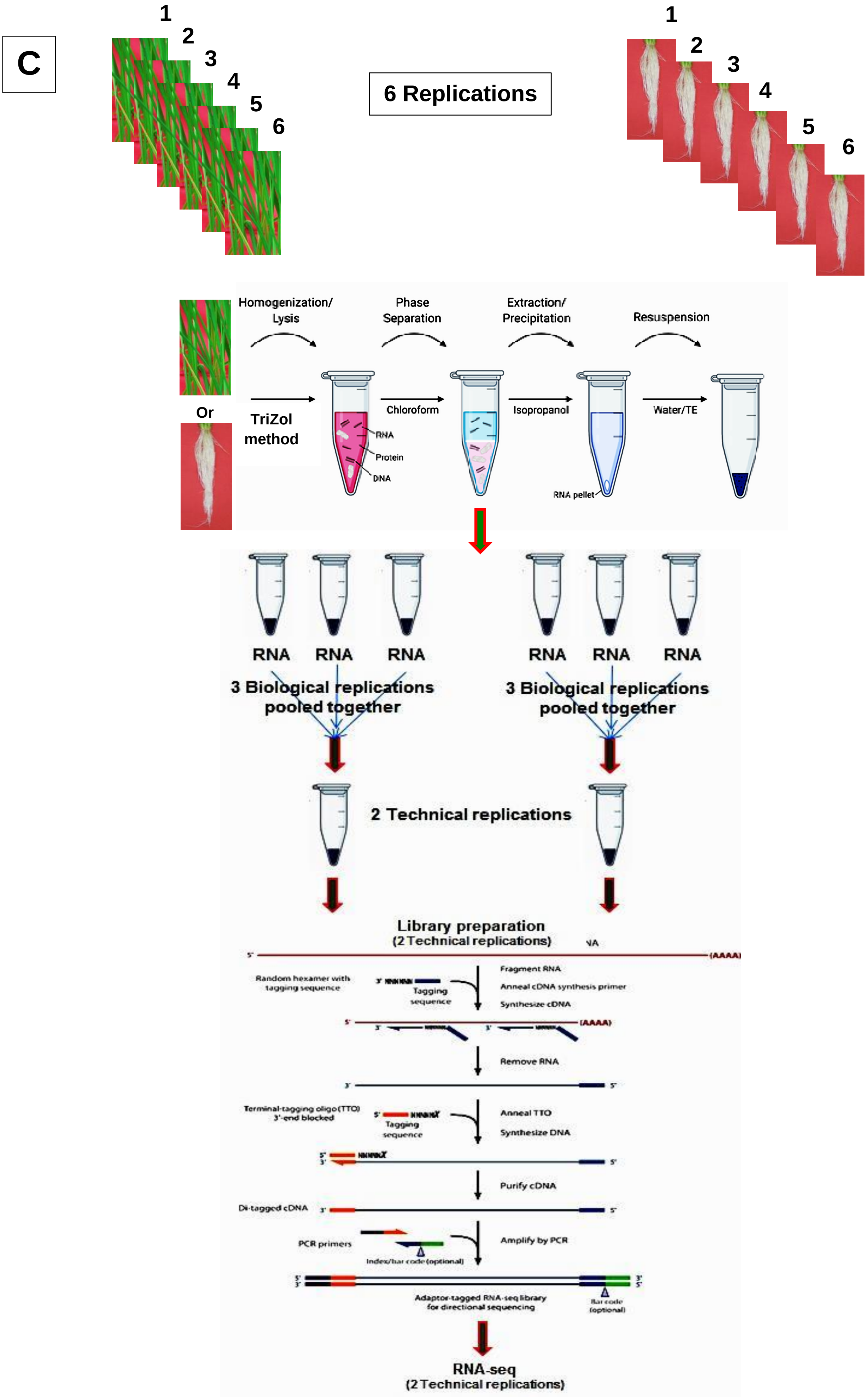

Supplement: Supplementary file 14 [file DataSheet_1.pdf]
