# Supplementary material for: Molecular basis of genetic plasticity to varying environmental conditions on growing rice by dry/direct-sowing and exposure to drought stress: Insights for DSR varietal development
Source: Front Plant Sci. 2022 Oct 24;13:1013207. doi: 10.3389/fpls.2022.1013207 (PMC9638133; doi:10.3389/fpls.2022.1013207)

Supplementary Figure S1: Representative picture of Nagina 22 and IR 64 cultivars subjected to drought stress at panicle initiation (reproductive) stage. The stress was imposed by withholding irrigation until the soil moisture content (SMC) reduced to ~6% and relative water content (RWC) of leaf came down to ~58% in the drought treated pots/plants compared to ~24% SMC and ~72% RWC for the control pots/plants.

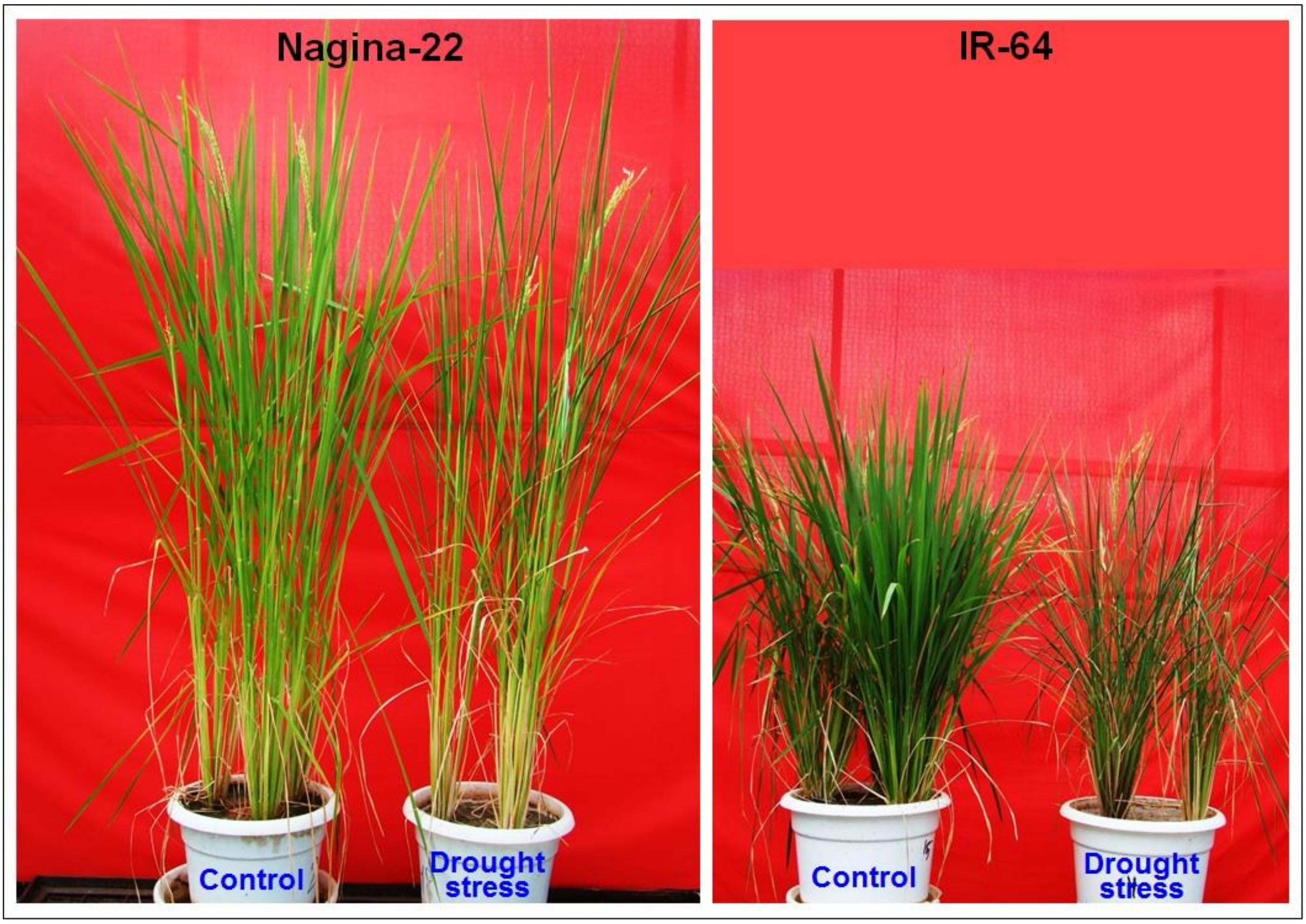

Supplementary Figure S2: Effect of the method of planting on growth and vigor of 28-days-old rice [IR 64 and Nagina 22 (N 22)] seedlings. (A) Rice seedlings grown by dry/direct-sowing in the main field, (B) the seedlings grown in nursery for transplantion.

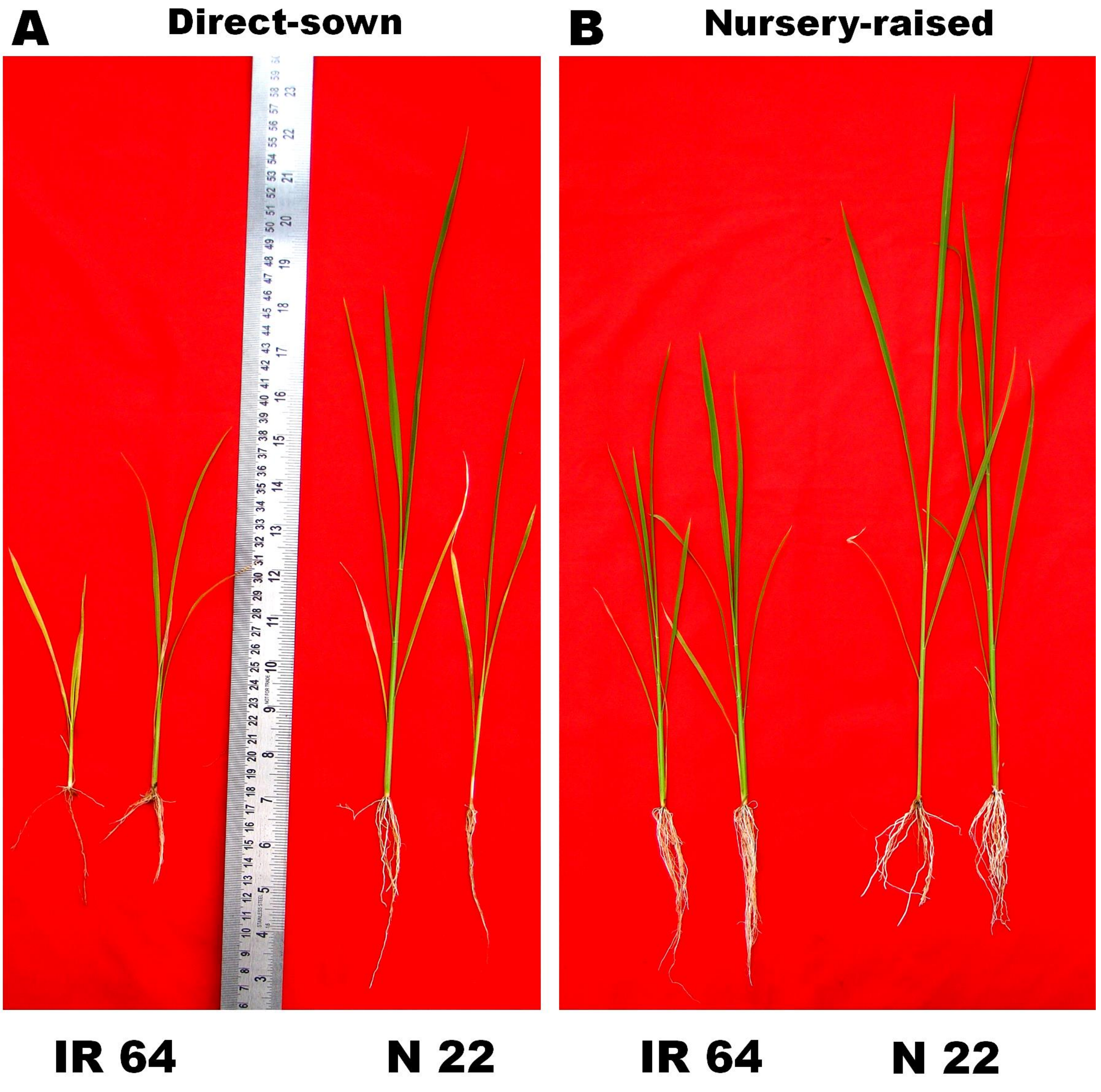

Supplementary Figure S3: Gene ontology (GO) analysis of under-represented biological processes in leaf of direct-sown over transplanted rice cultivars under drought stress. (A) Under-represented GO terms in the leaf of IR 64, and (B) under-represented GO terms in the leaf of Nagina 22 rice cultivar.

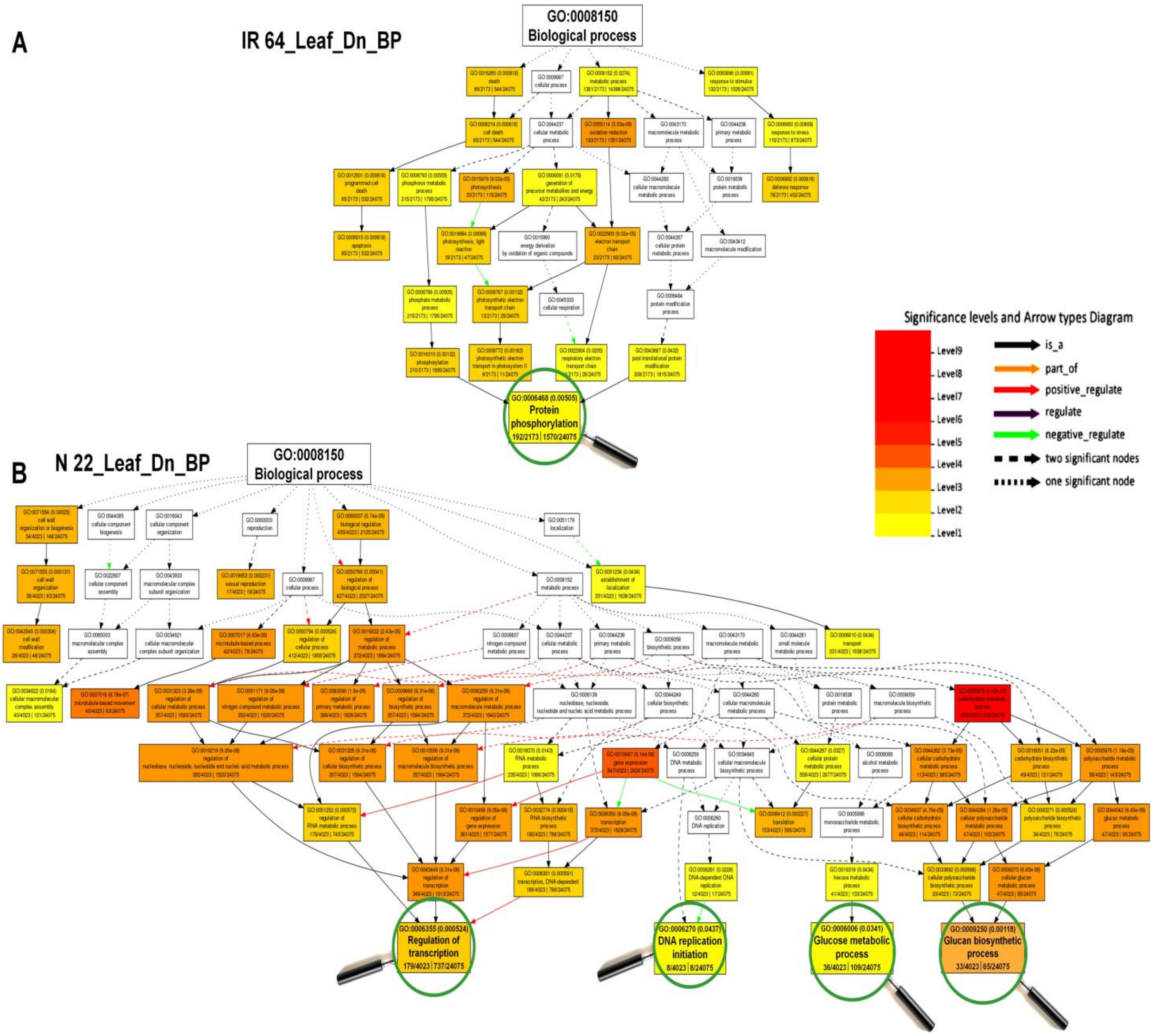

Supplementary Figure S4: Gene ontology (GO) analysis of enriched biological processes in root of direct-sown over transplanted rice cultivars under drought stress. (A) Enriched GO terms in root of IR 64, and (B) enriched GO terms in root of Nagina 22 rice cultivar.

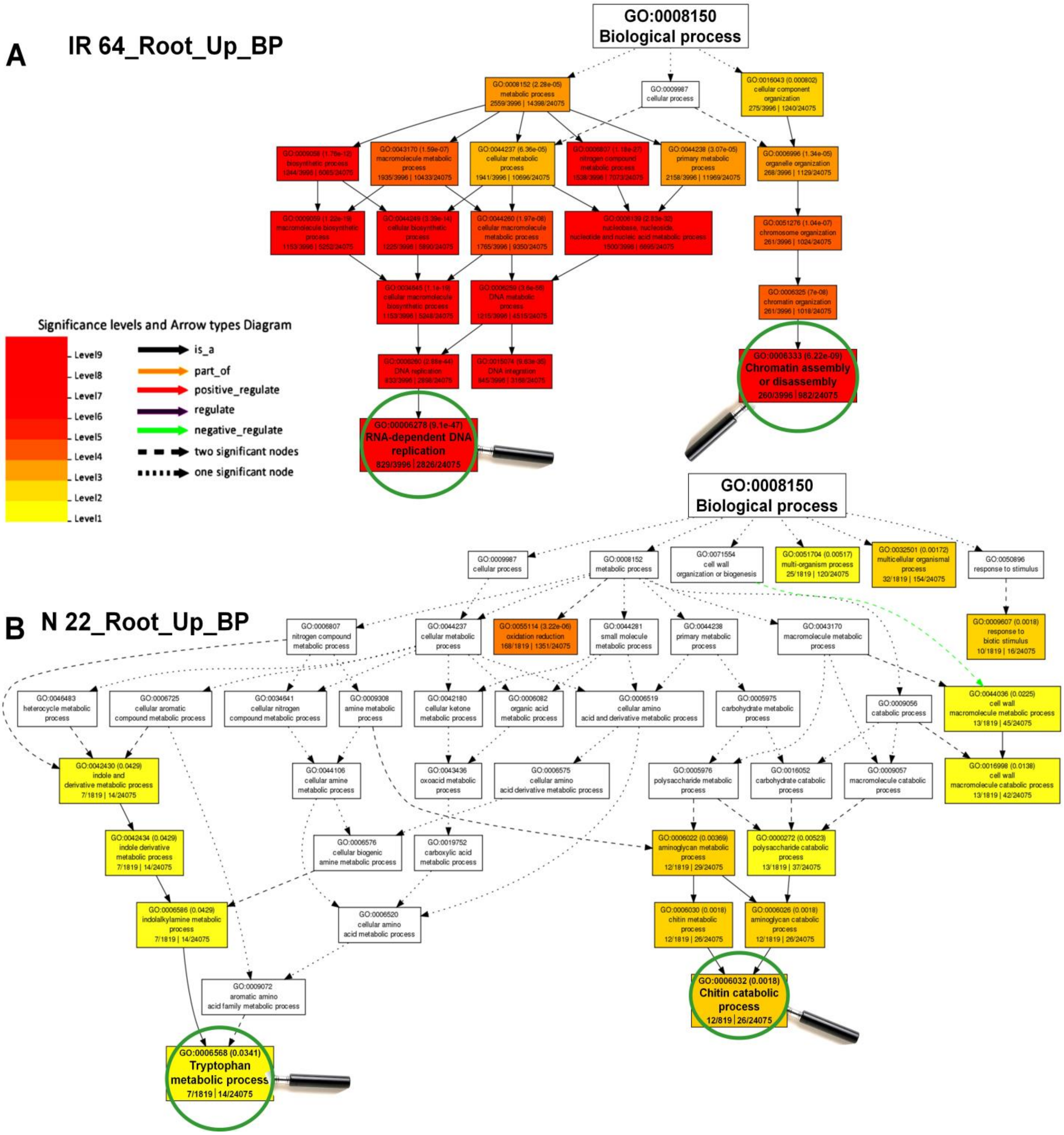

Supplementary Figure S5: Gene ontology (GO) analysis of under-represented biological processes in root of direct-sown over transplanted rice cultivars under drought stress. (A) Under-represented GO terms in root of IR 64, and (B) under-represented GO terms in root of Nagina 22 rice cultivar.

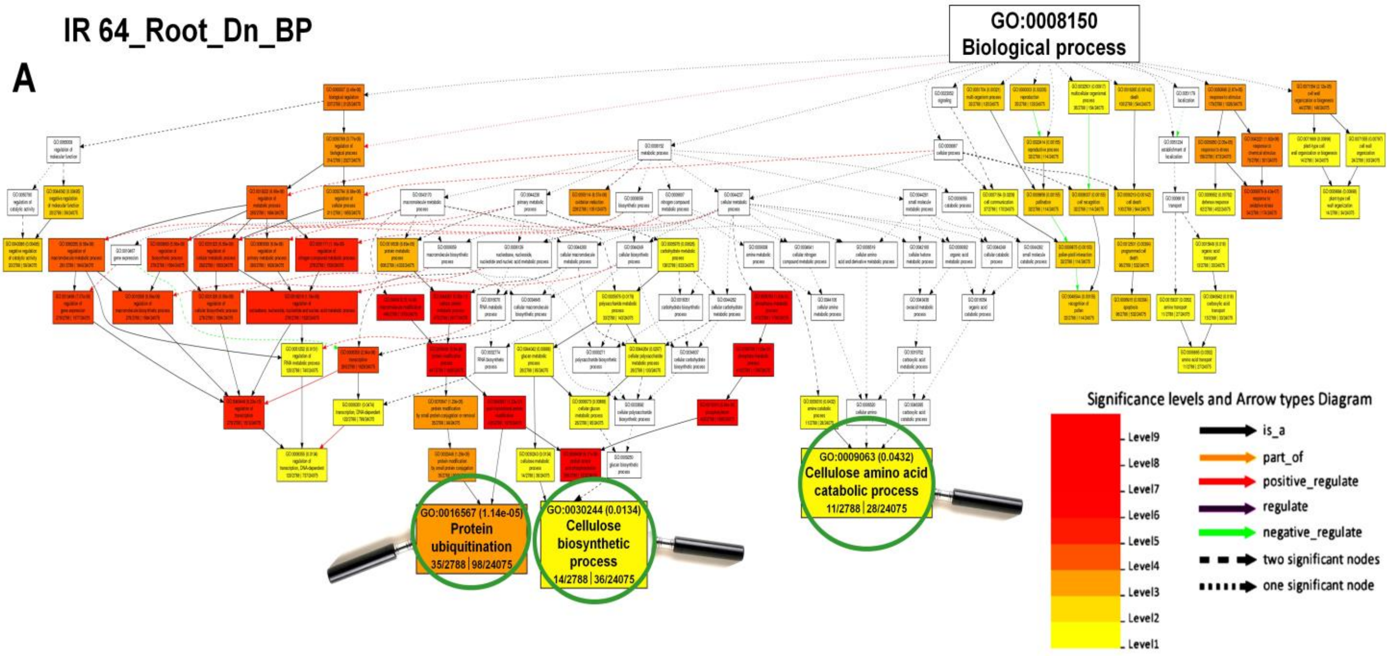

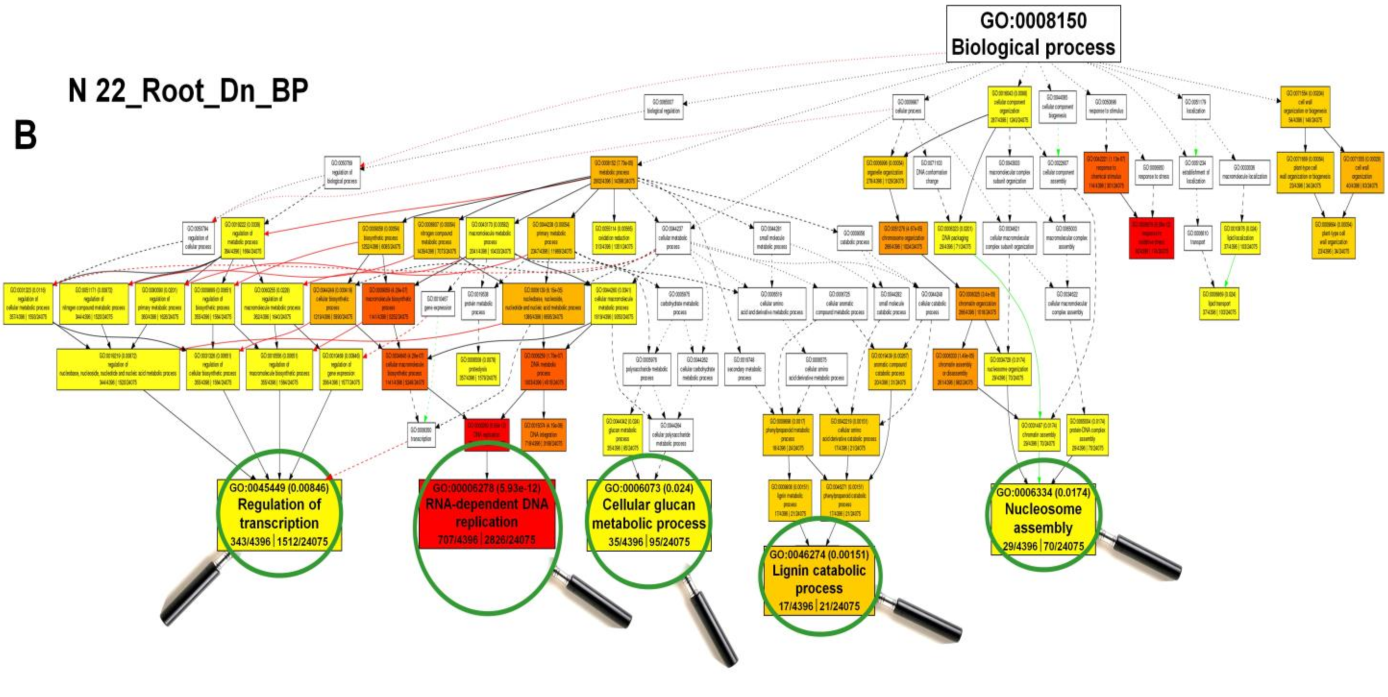

Supplementary Figure S6: Pathway enrichment analysis on direct-sowing imposed with drought stress in leaf of rice cultivars. (A) Under-represented process/pathway in the leaf of N 22, (B) under-represented process/pathway in IR 64 leaf, (C) over-represented process/pathway in N 22 leaf, and (D) over-represented process/pathway in the leaf of IR 64. Y-axis shows the name of the process/pathway, and the X-axis shows fold enrichment. Dot-size represents the number of genes and the color indicates the FDR value.

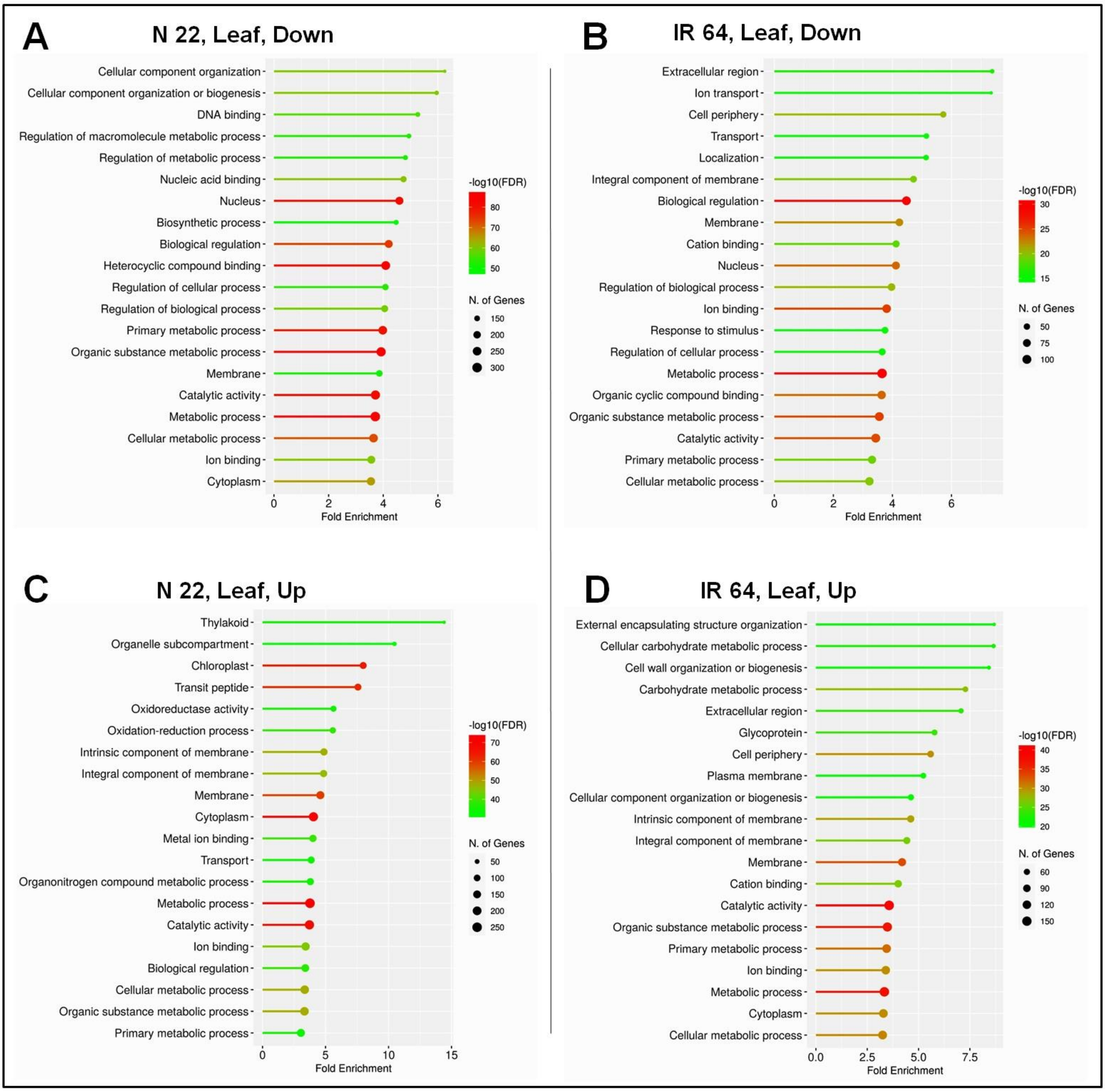

Supplementary Figure S7: Differentially expressed genes (DEGs) in rice cultivars [IR 64 and Nagina 22 (N 22)] on direct-sowing over transplanting (without drought stress) in (A) Leaf and (B) Root. Leaf and root tissues were collected at panicle-initiation stage of the plant for RNA-seq analysis.

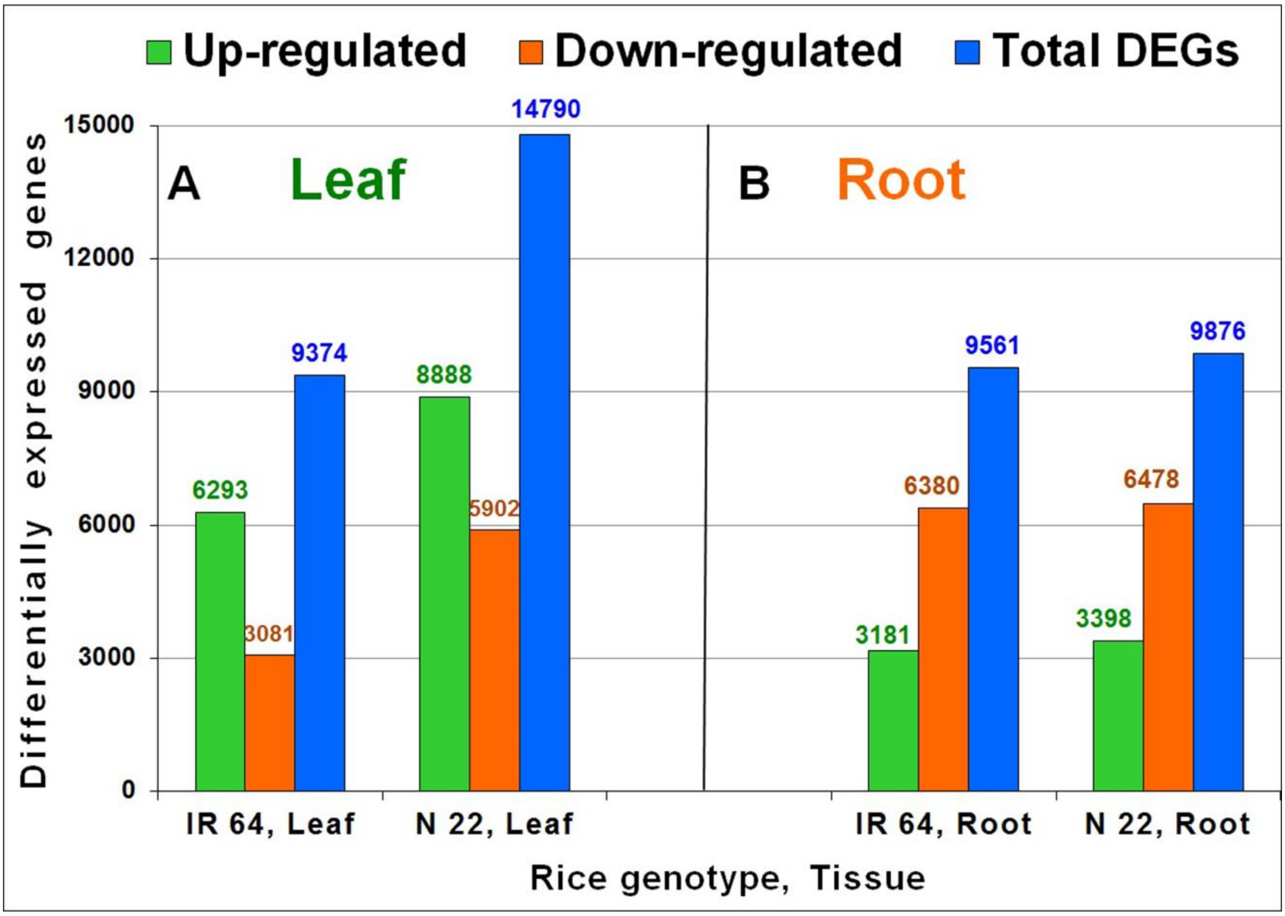

Supplement: Supplementary file 15 [file DataSheet_2.pdf]
